# Discovery and validation of a prognostic proteomic signature for tuberculosis progression: A prospective cohort study

**DOI:** 10.1371/journal.pmed.1002781

**Published:** 2019-04-16

**Authors:** Adam Penn-Nicholson, Thomas Hraha, Ethan G. Thompson, David Sterling, Stanley Kimbung Mbandi, Kirsten M. Wall, Michelle Fisher, Sara Suliman, Smitha Shankar, Willem A. Hanekom, Nebojsa Janjic, Mark Hatherill, Stefan H. E. Kaufmann, Jayne Sutherland, Gerhard Walzl, Mary Ann De Groote, Urs Ochsner, Daniel E. Zak, Thomas J. Scriba, ACS and GC6–74 cohort study groups

**Affiliations:** 1 South African Tuberculosis Vaccine Initiative, Division of Immunology, Department of Pathology and Institute of Infectious Disease and Molecular Medicine, University of Cape Town, Cape Town, South Africa; 2 SomaLogic, Inc., Boulder, Colorado, United States of America; 3 Center for Infectious Disease Research, Seattle, Washington, United States of America; 4 Max Planck Institute for Infection Biology, Berlin, Germany; 5 Medical Research Council Unit, The Gambia at the London School of Hygiene and Tropical Medicine, Fajara, The Gambia; 6 DST-NRF Centre of Excellence for Biomedical TB Research and MRC Centre for TB Research, Division of Molecular Biology and Human Genetics, Faculty of Medicine and Health Sciences, Stellenbosch University, Tygerberg, South Africa; John Hopkins University, UNITED STATES

## Abstract

**Background:**

A nonsputum blood test capable of predicting progression of healthy individuals to active tuberculosis (TB) before clinical symptoms manifest would allow targeted treatment to curb transmission. We aimed to develop a proteomic biomarker of risk of TB progression for ultimate translation into a point-of-care diagnostic.

**Methods and findings:**

Proteomic TB risk signatures were discovered in a longitudinal cohort of 6,363 *Mycobacterium tuberculosis*-infected, HIV-negative South African adolescents aged 12–18 years (68% female) who participated in the Adolescent Cohort Study (ACS) between July 6, 2005 and April 23, 2007, through either active (every 6 months) or passive follow-up over 2 years. Forty-six individuals developed microbiologically confirmed TB disease within 2 years of follow-up and were selected as progressors; 106 nonprogressors, who remained healthy, were matched to progressors. Over 3,000 human proteins were quantified in plasma with a highly multiplexed proteomic assay (SOMAscan). Three hundred sixty-one proteins of differential abundance between progressors and nonprogressors were identified. A 5-protein signature, TB Risk Model 5 (TRM5), was discovered in the ACS training set and verified by blind prediction in the ACS test set. Poor performance on samples 13–24 months before TB diagnosis motivated discovery of a second 3-protein signature, 3-protein pair-ratio (3PR) developed using an orthogonal strategy on the full ACS subcohort. Prognostic performance of both signatures was validated in an independent cohort of 1,948 HIV-negative household TB contacts from The Gambia (aged 15–60 years, 66% female), longitudinally followed up for 2 years between March 5, 2007 and October 21, 2010, sampled at baseline, month 6, and month 18. Amongst these contacts, 34 individuals progressed to microbiologically confirmed TB disease and were included as progressors, and 115 nonprogressors were included as controls. Prognostic performance of the TRM5 signature in the ACS training set was excellent within 6 months of TB diagnosis (area under the receiver operating characteristic curve [AUC] 0.96 [95% confidence interval, 0.93–0.99]) and 6–12 months (AUC 0.76 [0.65–0.87]) before TB diagnosis. TRM5 validated with an AUC of 0.66 (0.56–0.75) within 1 year of TB diagnosis in the Gambian validation cohort. The 3PR signature yielded an AUC of 0.89 (0.84–0.95) within 6 months of TB diagnosis and 0.72 (0.64–0.81) 7–12 months before TB diagnosis in the entire South African discovery cohort and validated with an AUC of 0.65 (0.55–0.75) within 1 year of TB diagnosis in the Gambian validation cohort. Signature validation may have been limited by a systematic shift in signal magnitudes generated by differences between the validation assay when compared to the discovery assay. Further validation, especially in cohorts from non-African countries, is necessary to determine how generalizable signature performance is.

**Conclusions:**

Both proteomic TB risk signatures predicted progression to incident TB within a year of diagnosis. To our knowledge, these are the first validated prognostic proteomic signatures. Neither meet the minimum criteria as defined in the WHO Target Product Profile for a progression test. More work is required to develop such a test for practical identification of individuals for investigation of incipient, subclinical, or active TB disease for appropriate treatment and care.

## Author summary

**Why was this study done?:**

- Tuberculosis (TB) is currently the leading cause of death by an infectious disease, yet diagnosis of TB is still hampered by poor tools that require a sputum sample.
- An accurate, affordable, and easy-to-use diagnostic test would allow targeted antibiotic treatment before symptoms develop and the person becomes infectious, thus providing an opportunity to curb transmission and halt the global epidemic.

**What did the researchers do and find?:**

- In this study, we sought to develop a blood test that can predict if a healthy individual is likely to progress to active TB disease before clinical symptoms manifest.
- We analyzed plasma from healthy South African adolescents who were followed over 2 years. By comparing abundance of over 3,000 different plasma proteins from individuals who developed TB disease and others who remained healthy, we identified 2 biomarkers that comprised combinations of either 3 or 5 proteins and predicted onset of TB a year before traditional diagnosis was possible.
- The protein biomarkers were validated for accuracy in an independent cohort of individuals from The Gambia.

**What do these findings mean?:**

- To our knowledge, these are the first validated protein biomarkers with prognostic value for TB; however, neither meet the minimum performance criteria as set out by WHO for a TB progression test.
- More work is required to improve the performance of such tests for practical identification of individuals for investigation of incipient, subclinical, or active TB disease.

## Introduction

Global efforts to control the tuberculosis (TB) epidemic depend on new, more efficacious TB vaccines and drugs in addition to better diagnostic tests to accurately diagnose those with TB disease. Earlier identification of individuals during incipient or subclinical stages of TB disease progression holds great promise for targeted preventive therapy, which may provide a strategy to curb onward transmission of *M*. *tuberculosis*. Such a strategy requires prognostic tests that can accurately identify those at risk of TB disease before the onset of symptoms and further transmission. In 2017, 10 million cases of TB and 1.6 million deaths (more than any other infectious agent) were reported [1–3]. It is estimated that up to 40% of these TB cases are missed and thus not treated, highlighting the limitations of current diagnostic strategies and emphasizing the need for better, faster, and more tractable diagnostic tests [2].

In people with asymptomatic *M*. *tuberculosis* infection, the infecting organisms are primarily contained within lung granulomas and/or draining lymph nodes, making direct detection of the bacterium virtually impossible. However, host signals in the blood compartment, such as inflammatory markers, have been shown to reflect the host–pathogen interactions at the site of disease, which can be used to identify those who are progressing from *M*. *tuberculosis* infection to active TB disease. For example, we validated blood transcriptomic signatures of TB risk that identified those who progressed to active disease up to 18 months before TB diagnosis [4,5]. Although these RNA-based biomarkers show promise, measurement of plasma proteins is more amenable to development of point-of-care tests, as exemplified by lateral flow tests based on capillary blood collected by needle prick. Indeed, profound changes in abundance of many plasma proteins have been reported in TB patients, and we and others have described protein-based diagnostic TB signatures [6–9]. Further, by measuring kinetic changes in plasma proteins in TB progressors, we observed that proteins involved in inflammatory pathology, tissue repair, matrix-remodeling, elevated interferon responses, and activation of the complement pathway revealed stages of TB disease progression [10]. Similarly, Esmail and colleagues showed that HIV-infected individuals with subclinical TB had elevated plasma levels of immune complexes and blood signatures of complement activation [11].

In this study, we proposed to identify and validate parsimonious proteomic signatures of TB disease risk. We measured >3,000 proteins by multiplexed slow off-rate modified DNA aptamers (SOMAmers) in plasma from *M*. *tuberculosis*-infected progressors and nonprogressors and identified 2 proteomic signatures of TB progression, which were validated in an independent cohort.

## Methods and materials

### Participant selection

#### Discovery cohort

The discovery cohort comprised a subset of 6,363 healthy South African adolescents, aged 12–18 years, who were enrolled into the Adolescent Cohort Study (ACS) between July 6, 2005 and April 23, 2007 [4,12]. The study protocols were approved by the Human Research Ethics Committee of the University of Cape Town (045/2005). Adolescents whose parents or legal guardians provided written, informed consent and who provided written, informed assent themselves were eligible for enrollment. Participants were followed for 2 years, with 50.9% (3,236 of 6,363) assessed every 6 months after enrollment, and the other 49.1% (3,127 of 6,363) at baseline and at 2 years (passive follow-up group). These 2 follow-up strategies were applied to determine whether a passive follow-up design would allow efficient TB case finding in this setting in preparation for large vaccine trials [13]. At enrollment and at each visit, clinical data were collected, and plasma from heparin containing Cell Preparation Tubes (CPT, BD Biosciences) was collected, stored at −80°C, and later used for proteomic analysis. Only adolescents with immunological sensitization to *M*. *tuberculosis* were included in the analysis, diagnosed by a positive QuantiFERON TB Gold In-tube assay, a positive tuberculin skin test (TST), or both, as previously described [4]. Further details about the prevalence of *M*. *tuberculosis* infection and disease in the ACS have been published [12,13], while clinical and epidemiological attributes and the selection of progressors and nonprogressors are in S2 Text and S1 Table. According to South African policy, adolescents positive on these tests were not offered therapy to prevent TB disease [14].

During follow up, 46 individuals developed intrathroracic TB disease, diagnosed by either 2 consecutive sputum smears positive on microscopy for acid-fast bacilli or 1 positive sputum culture confirmed as *M*. *tuberculosis* complex (Mycobacterial growth indicator tube, BD BioSciences). These TB “progressors” were each matched to 2 “nonprogressors” (individuals who did not develop TB disease) during follow-up by accounting for age, gender, ethnicity, school of attendance, and prior history of TB disease. Adolescents who were known to be HIV-infected and those who developed TB disease within 6 months of ACS enrollment were excluded from the progressor/nonprogressor subcohort on the basis that they may represent individuals with active but as yet asymptomatic TB disease. To our knowledge, participants did not have any other underlying diseases.

Discovery of the TB Risk Model 5 (TRM5) signature was initially performed on a partition of 67% of the ACS progressor/nonprogressor cohort, while the remaining 33% was held back as a blinded test set. As reported in the Results section, application of the TRM5 signature to the ACS test set provided evidence for the viability of a predictive proteomic signature. However, the TRM5 signature did not significantly discriminate between progressor and nonprogressor samples collected more than 1 year before TB diagnosis. Our previous work on transcriptomic signatures, which showed that a 16-gene mRNA signature allowed significant discrimination between samples from progressors and nonprogressors at time points more than 12 months before TB diagnosis [4], suggested that a larger training set, which incorporated more progressor samples collected 13–24 months before TB, may allow discovery of a superior signature. We therefore sought to refine the proteomic signature using the combined ACS training and test set under the hypothesis that a larger training set could help bolster performance more than a year from diagnosis. Having demonstrated predictive capacity using the TRM5 signature, at this point we also sought to construct a maximally parsimonious signature that could more simply be translated into a point-of-care test. We therefore constructed the 3-protein pair-ratio (3PR) signature from the entire ACS progressor/nonprogressor cohort, using a methodology designed to lead to parsimonious signatures that are robust to translation from an omics to a targeted platform.

The script for computing the TRM5 signature is available from SomaLogic upon request. The script for computing for the 3PR signature is available at BitBucket (https://bitbucket.org/satvi/3pr). Both signatures were validated by blind prediction in the validation cohort.

#### Validation cohort

The validation cohort comprised a subset of Gambian participants of the Grand Challenges 6–74 (GC6–74) project, as previously described [4,5,15]. Briefly, between March 5, 2007 and October 21, 2010, household contacts of TB cases were longitudinally followed for up to 2 years, with assessments at baseline, at 6 months, and at 18 months. Immunological sensitization to *M*. *tuberculosis* was performed by TST. TB progressors who developed microbiologically confirmed pulmomary TB during follow-up were retrospectively identified and matched 1:4 to healthy nonprogressors. Individuals in whom TB disease developed within 3 months of baseline were excluded. Blood and plasma were collected in lithium heparin tubes (Becton Dickinson) at enrollment, month 6, and month 18 of the GC6–74 project, and 254 plasma samples from 34 progressors and 115 nonprogressors were included for validation. Further details about clinical and epidemiological attributes and the selection of progressors and nonprogressors are provided in S2 Text and S2 Table. Participants provided written, informed consent, and the protocols were approved by the Joint Medical Research Council and Gambian Government ethics review committee, Banjul, The Gambia (SCC.1141vs2).

#### Multiplex proteomic detection

Proteomic analysis was performed using SOMAscan, a proprietary multiplexed assay to detect the abundance of 3,040 proteins recognized by slow off-rate modified aptamer (SOMAmer) reagents, as previously described [16]. Samples from the validation cohort were assayed using a custom SOMAscan assay with smaller content, as described below. Plasma was analyzed at 3 different dilutions (0.005%, 1%, and 40% of original plasma) using separate SOMAmer reagent mixes to accommodate high-, medium-, and low-abundant plasma proteins [16]. Quality control procedures used control aptamers for data normalization, hybridization control probes to measure hybridization efficiency, and calibration samples to control inter- and intra-assay variability. Assay quality control and data standardization was performed following SOMAscan data normalization standard operating procedures [17]. Briefly, calibration samples were used to control for assay variability, and hybridization normalization is used to remove potential biases introduced by differential hybridization efficiency within and across assay runs.

#### Focused hybridization arrays for validation study

A customized, focused panel array was designed based on findings from the discovery phase of the project [4,10] and was used for validation sample sets. The strategy and analysis plan for signature discovery, verification, and validation is outlined in S2 Text, and a schematic of the approach is shown in S1 Fig. This panel consisted of 150 SOMAmers common to the >3,000-plex discovery array, including targets for human, *M*. *tuberculosis*, and array normalization proteins.

The 254 GC6–74 validation samples were assayed on the validation panel along with the standard SOMAscan calibration (*n* = 20), quality control (*n* = 12), and buffer samples (*n* = 12). In addition, 45 ACS samples from the discovery cohort (15 progressors), which were previously analyzed on the discovery assay, were included as bridging samples to assess and adjust for potential biases due to differences in the assay format or reagent changes since the discovery assays were performed. The slides for the focused array were manufactured by Applied Microarrays Inc. (Tempe, Arizona). Although custom SOMAmer mixes were prepared for the smaller content hybridization arrays, no other assay format changes were introduced. Internal assay method development studies had previously established the performance of these smaller hybridization arrays.

#### Blinding procedure

Samples from the ACS test set (33% of the progressor and nonprogressor cohort) were blinded through nonsequential randomly generated codes, held in a locked database by the project manager. Unblinding occurred in a staged manner; once models and scripts were locked down and each partner institute had validated that results obtained on the blinded set were identical and reproducible, an interim analysis of longitudinally collected samples from the same participants were performed without revealing case/control status. Subsequently, progressor and nonprogressor status were unblinded to all sites simultaneously and performance of models were independently calculated and confirmed.

All 254 GC6–74 plasma samples were deidentified and provided nonsequential randomly generated codes, which were held in a locked database by the project manager. Unblinding of samples, matched participants, and progressor/nonprogressor status occurred simultaneously. A detailed description of the analysis strategy for signature discovery, verification, and validation is available in S2 Text.

#### Statistical analysis and model development

Proteomic data were log_10_ transformed to stabilize the variance and reduce heteroscedasticity. Of the 3,040 proteins, 2,872 passed quality control in both the ACS training and test set assays (S3 and S4 Tables and S2 Text). The nonparametric Kolmogorov–Smirnov test was used to identify proteins differentially expressed between progressors and nonprogressors. In addition, we sought “responsive proteins,” those with differential temporal responses across time, using the nonparametric Mack–Wolfe [18] test with discrete 6-month bins to identify proteins with time-varying expression levels in either progressors or nonprogressors (see S2 Text for more detail). Differentially expressed proteins for hypothesis generation and for multivariate predicitive model building were identified using 1% and 5% Benjamini–Hochberg (BH) corrected false discovery rates, respectively.

The TRM5 is a Mahalanobis distance classifier (see S2 Text for more detail). Model parameters were estimated using protein measurements from the nonprogressors only (the model functions as an anomaly detector), and samples with protein levels that are anomalous with respect to the joint distribution of model proteins in the “nonprogressor” class were considered progressors. All possible combinations of 1-, 2-, 3-, 4-, and 5-protein models were fit, and performance was estimated using 5 rounds of 5-fold cross-validation, with a progression time-weighted AUC measure as the cost function.

The 3-protein model of risk for TB disease progression was developed by applying the Pair Ratios algorithm to the ACS progressor and nonprogressor cohort from the combined training and test sets, in a variation on the pairwise approach used to discover the 16-gene ACS COR and the RISK4 signatures [4,5,19,20]. The Pair Ratios algorithm results in an ensemble of protein pairs, which each provide a risk score for each sample (see S2 Text for detailed methods). The final model score for each sample is then computed as the average over the scores generated from each pair. The final 3-protein signature was selected based on a balance between signature size and performance. Out of all 3-protein signatures, the 3PR signature optimally stratified the training set, and 3PR performance did not significantly differ statistically from the optimal larger signatures. Because the 3PR signature is ratiometric and involves only 3 proteins, it is ideally suited for translation to a targeted platform.

After unblinding, an ANOVA model was used to assess differences in distributions of protein signature scores between the ACS training set control samples (from which the TRM5 model was fit) and the GC6–74 control samples.

## Results

### Sample availability and distribution

**Fig 1:**
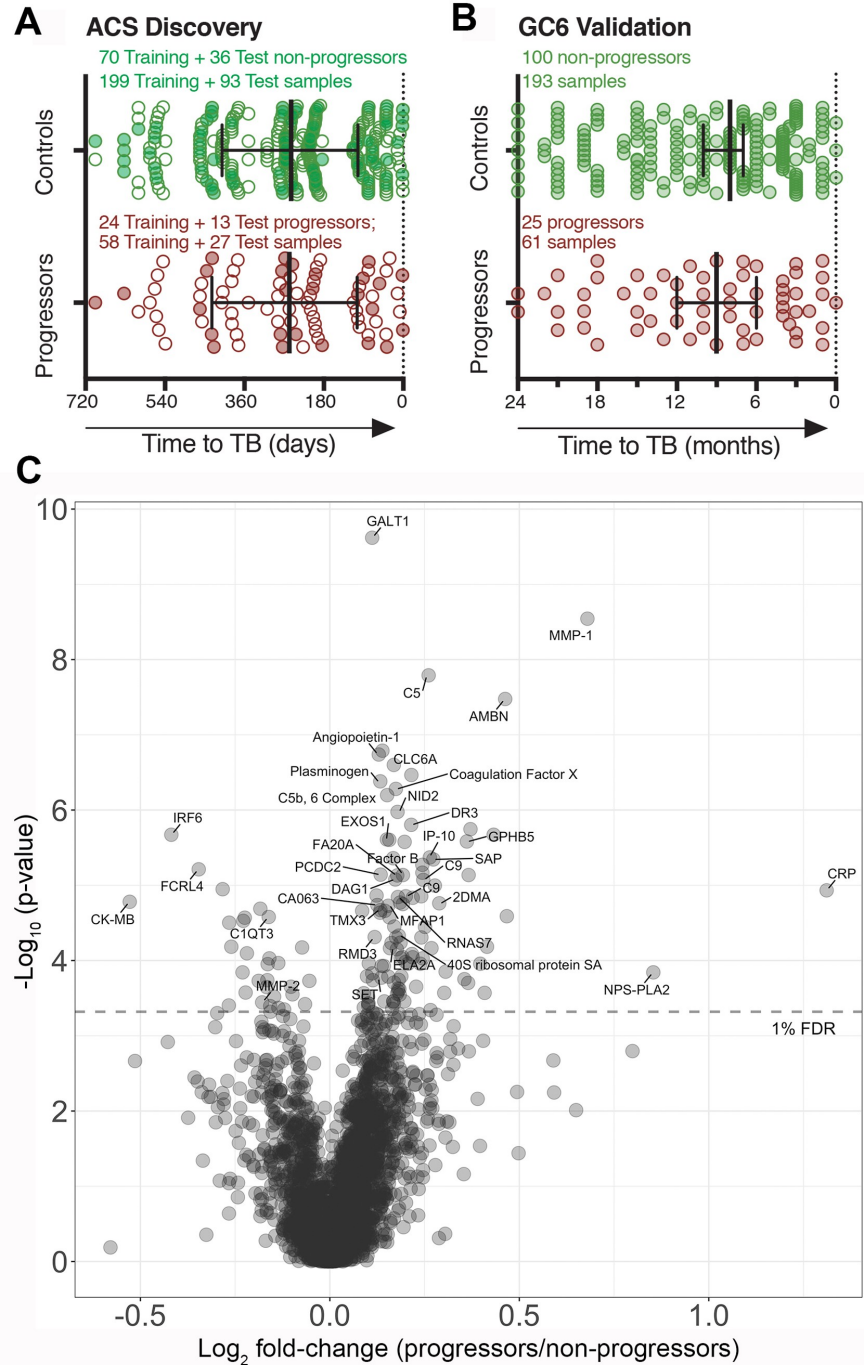
Sample distribution and relative differences in protein abundance between progressors and nonprogressors. (A) Distribution of progressor and nonprogressor samples from the discovery training and test set of South African adolescents and (B) progressor and nonprogressor samples from Gambian household contacts of TB cases used for validation. Progressor and nonprogressor samples are represented by filled and open dots, respectively. The x-axis indicates time of prospective sample collection before the diagnosis of active TB disease. Nonprogressor samples were matched to progressors, as previously described [4,5], and aligned with time to TB diagnosis. (C) Volcano plot of 2,872 proteins from a univariate KS analysis comparing all TB progressor samples and all nonprogressor controls. The negative log_10_-transformed *P* values versus the log_2_ of the median TB RFU value over the median control RFU value. A value of 1 on the horizontal axis corresponds to a 2-fold change in RFU. Protein abundance data are in S4 and S6 Tables, and proteins ranked according to their differential abundances are in S5 Table (training set), S7 Table (training and test set), and S2 Text. KS, Kolmogorov–Smirnov; RFU, raw fluorescence unit; TB, tuberculosis.

Plasma samples were available for 37 progressors and 106 nonprogressors from the ACS and were primarily distributed between 1–18 months before TB diagnosis (Tables 1 and S1 and Fig 1A and S2 Text). Participants were randomly split into training and test sets for TRM5 signature discovery at a ratio of 2:1 (Fig 1A). Longitudinally collected samples from each participant were retained in each set and evaluated to ensure sufficient distribution of progressor samples in each 6-month time window approaching the diagnosis of TB disease.

**Table 1:** Participant demographics for the progressor and nonprogressor cohorts with available plasma samples in ACS training and test sets and the GC6–74 validation cohort.

	Participants	Age	Male	Black African	Cape Mixed Ancestry	Prior TB
	*n*	Mean, (min–max)	*n*, (%)	*n*, (%)	*n*, (%)	*n*
**ACS Training**						
**Progressors**	24	15.83 (13–18)	6 (25%)	0 (0%)	24 (100%)	3 (12.5%)
**Nonprogressors**	70	15.67 (13–18)	20 (28.57%)	4 (5.71%)	66 (94.29%)	10 (14.29%)
**ACS Test**						
**Progressors**	13	15.15 (12–18)	3 (23.08%)	1 (7.69%)	12 (93.31%)	2 (15.38%)
**Nonprogressors**	36	15.58 (13–18)	17 (47.22%)	5 (13.89%)	31 (86.11%)	6 (16.67%)
**GC6–74 Validation Cohort**						
**Progressors**	34	26.91 (15–56)	15 (44.12%)	34 (100%)	0 (0%)	N/A*
**Nonprogressors**	115	27.27 (15–60)	52 (45.22%)	115 (100%)	0 (0%)	N/A*

**Abbreviations:** ACS, Adolescent Cohort Study; GC6–74, Grand Challenges 6–74; N/A, not available;TB, tuberculosis.

*Prior TB was an exclusion criterion in the GC6–74 study.

Similarly, plasma samples from 34 progressors and 115 nonprogressors from the Gambian GC6–74 cohort were available for blind validation and distributed between 1–24 months before TB diagnosis [4,5] (Tables 1 and S2 and Fig 1B and S2 Text). A sample-by-sample hybridization normalization was first applied to control for differential hybridization of SOMAmers to the readout microarrays. An intraplate median signal normalization was then applied to control for bulk signal differences between samples. Finally, between-plate signal differences were corrected by calibrating each plate using replicate calibrator samples. For the GC6–74 samples, an additional 45 bridging samples were selected from the ACS cohort and were used to bring the distributions into alignment using a linear transformation.

Protein abundance data are presented in S4 and S6 Tables and S2 Text.

### Identification of differentially expressed proteins in TB progressor and nonprogressor plasma samples

To identify host proteins with differential abundance, we compared all 197 nonprogressor plasma samples with 56 progressor samples from the ACS training set. One hundred thirty-five proteins were found to be different at a 1% Benjamini–Hochberg False Discovery Rate (bhFDR). Of these, 105 proteins were significantly more abundant and 30 proteins less abundant in progressors relative to nonprogressors (Fig 1C and S5 Table and S2 Text). The most differentially abundant protein between progressors and nonprogressors was Galactose-1-phosphate uridyl transferase 1 (GALT-1, log_2_ fold change = 0.112; *P* = 2.40 x 10^−10^; S5 Table and S2 Text), which is involved in galactose metabolism pathways, followed by Matrix Metalloproteinase 1 (MMP-1, log_2_ fold change = 0.680; *P* = 2.86 x 10^−9^), both of which were more abundant in progressors than nonprogressors. The protein found to be most abundant in progressors relative to nonprogressors was the acute-phase marker C-reactive protein (CRP, log_2_ fold change = 1.31; *P* = 1.17 x 10^−5^). The protein found at lowest levels in progressors relative to nonprogressors was Creatine Kinase type M/type B (CK-MB, log_2_ fold change = −0.528; *P* = 1.66 x 10^−5^).

### Discovery of a 5-protein signature of risk in the ACS training cohort

**Fig 2:**
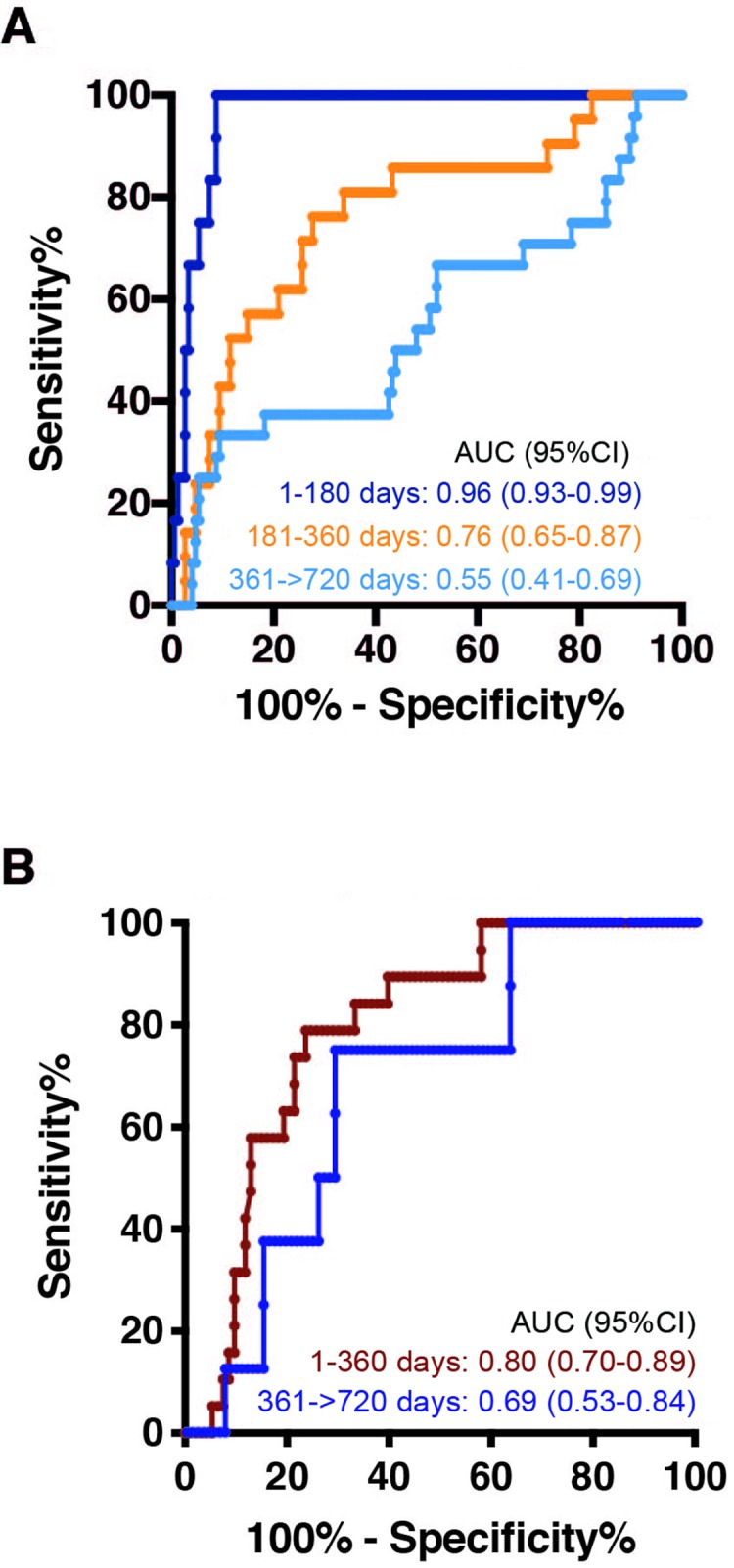
Receiver operator characteristic AUC analysis of the TRM5 signature for (A) ACS training set and (B) test set progressor and nonprogressor plasma samples, stratified by the time interval of each prospectively collected sample before the date of TB disease diagnosis. ACS, Adolescent Cohort Study; AUC, area under the curve; TB, tuberculosis; TRM5, TB Risk Model 5.

Amongst all possible signatures with 1, 2, 3, 4, or 5 proteins, the signature with the highest AUC in cross-validation on the ACS training set was a 5-protein signature called TRM5, consisting of complement factor C9, insulin-like growth factor-binding protein 2 (IGFBP-2); B-cell antigen receptor complex–associated protein (CD79A), Matrix-Remodeling Associated 7 protein (MXRA-7), and neuronal cell-adhesion molecule (NrCAM). TRM5 signature scores were higher in progressors than nonprogressors, and the signature readily discriminated progressor from nonprogressor samples collected 1 to 180 days before TB diagnosis (AUC 0.961; 95% CI 0.931–0.99, Fig 2A and Table 2). Prognostic performance decreased for samples collected between 181 and 360 days before TB diagnosis, with an AUC of 0.761 (95% CI 0.648–0.874, Fig 2A and Table 2). The TRM5 signature did not significantly discriminate between progressor and nonprogressor samples collected more than 1 year before TB diagnosis (AUC 0.55; 95% CI 0.414–0.691, Fig 2A and Table 2).

**Table 2:** Prognostic performance of the TRM5 and 3PR signatures on samples from the ACS (discovery) and GC6–74 (validation) cohorts.

	Time to TB (months)	No. progressor samples	AUC (95% CI)	*P*	Sensitivity (95% CI)	Specificity (95% CI)*	Threshold
**TRM5**	**ACS Training**						
	0–12	33	0.84 (0.75–0.92)	N/A^#^	75.76 (57.74–88.91)	75.0 (67.22–81.75)	4.18
	0–6	12	0.96 (0.93–0.99)	N/A	100 (73.54–100)	75.0 (67.22–81.75)	4.18
	7–12	21	0.76 (0.65–0.87)	N/A	61.9 (38.44–81.89)	75.0 (67.22–81.75)	4.18
	13–>24	24	0.55 (0.41–0.69)	N/A	37.5 (18.8–59.41)	70.27 (62.21–77.5)	4.18
	**ACS Test**						
	0–12	19	0.80 (0.70–0.89)	<0.0001	78.95 (54.43–93.95)	75.27 (65.24–83.63)	4.28
	0–6	10	0.85 (0.75–0.96)	0.0003	90 (55.5–99.75)	75.27 (65.24–83.63)	4.28
	7–12	9	0.73 (0.62–0.85)	0.0208	66.67 (29.93–92.5)	75.27 (65.24–83.63)	4.28
	13–>24	8	0.69 (0.53–0.84)	0.076	37.5 (8.52–75.51)	75.27 (65.24–83.63)	4.28
	**GC6 Validation**						
	0–12	41	0.66 (0.56–0.75)	0.0016	48.78 (32.88–64.87)	75.0 (68.26–80.96)	16.45
	0–6	23	0.69 (0.57–0.82)	0.0026	60.87 (38.54–80.29)	75.0 (68.26–80.96)	16.45
	7–12	18	0.61 (0.48–0.74)	0.1146	33.33 (13.34–59.01)	75.0 (68.26–80.96)	16.45
	13–24	19	0.61 (0.47–0.75)	0.1179	36.84 (16.29–61.64)	75.0 (68.26–80.96)	16.45
**3PR**	**ACS Training + Test**						
	0–12	52	0.80 (0.74–0.86)	N/A^#^	75 (61.05–85.97)	70.69 (65.09–75.87)	0.51
	0–6	22	0.89 (0.84–0.95)	N/A	90.91 (70.84–98.88)	70.69 (65.09–75.87)	0.51
	7–12	30	0.72 (0.64–0.81)	N/A	63.33 (43.86–80.07)	70.69 (65.09–75.87)	0.51
	13–>24	32	0.71 (0.63–0.80)	N/A	57.14 (37.18–75.54)	70.69 (65.09–75.87)	0.51
	**GC6 Validation**						
	0–12	41	0.65 (0.55–0.75)	0.0022	46.34 (30.66–62.58)	75.0 (68.2–80.96)	0.28
	0–6	23	0.64 (0.50–0.78)	0.0266	47.83 (26.82–69.41)	75.0 (68.26–80.96)	0.28
	7–12	18	0.67 (0.55–0.79)	0.0194	44.44 (21.53–69.24)	75.0 (68.26–80.96)	0.28
	13–24	19	0.62 (0.48–0.76)	0.0873	42.11 (20.25–66.5)	75.0 (68.26–80.96)	0.28

**Abbreviations:** 3PR, 3-protein pair-ratio; ACS, Adolescent Cohort Study; GC6, Grand Challenge 6; TB, tuberculosis.

*Specificity has been set to 75% (or the closest possible value) based on the minimum performance criteria set out in the target product profile of WHO and FIND. The corresponding sensitivities and test threshold of each risk signature are reported.

^#^*P* values are not reported for model fit to the training cohorts.

### Verification of TRM5 signature on the ACS test cohort

To assess performance of the TRM5 signature on an unseen verification partition of the ACS progressors and nonprogressors, we applied it to blinded plasma samples from the ACS test set, comprising 13 progressors and 36 nonprogressors who were not included in the model discovery training set. The TRM5 signature discriminated progressor from nonprogressor samples spanning 1–720 days before TB with an AUC of 0.76 (95% CI 0.67–0.86, *P* < 0.001, Fig 2B), verifying the performance observed in the training set.

### Discovery of a 3PR signature of risk

**Fig 3:**
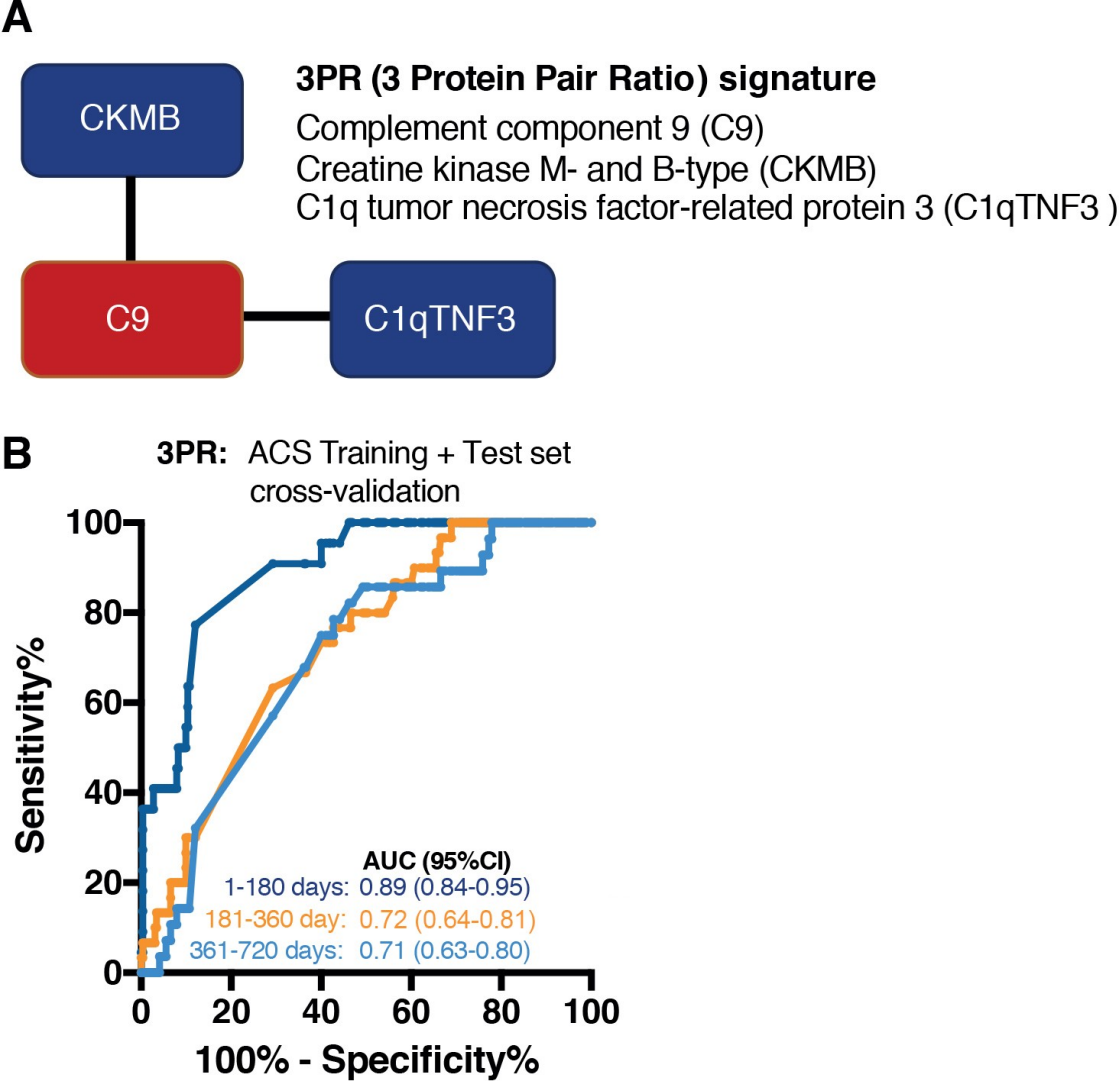
(A) Graphical representation of pairwise structure of the 3PR signature. Proteins that are expressed at higher levels in TB progressors, compared to nonprogressors, are shown in red. Proteins expressed at levels lower in progressors than nonprogressors are shown in blue. (B) Area under the receiver operator characteristic curve analysis of the 3PR signature for all ACS progressor and nonprogressor plasma samples, stratified by the time of each prospectively collected sample before the date of TB disease diagnosis. 3PR, 3-protein pair-ratio; ACS, Adolescent Cohort Study; TB, tuberculosis.

Our work on transcriptomic signatures showed that a 16-gene mRNA signature allowed significant discrimination between samples from progressors and nonprogressors at time points more than 12 months before TB diagnosis [4]. We therefore employed a different discovery approach that combined the ACS training and test sets to develop a signature that may provide better discrimination in samples collected more than a year before TB diagnosis. In this strategy, we also sought to make the signature as parsimonious as possible; we employed a pair-ratio strategy that incorporates a small ensemble of pairwise models, each comprising 1 protein with higher and 1 with lower abundance in progressors relative to nonprogressors. Using leave-one-out cross-validation, 3 proteins were selected, including C9 (higher in progressors than nonprogressors), CK-MB, and Complement C1q Tumor Necrosis Factor-Related Protein 3 (C1qTNF3/CTNFF3) (both lower in progressors than nonprogressors), which together formed the 3PR signature, an ensemble of 2 protein pairs (Fig 3A). Only 1 protein, C9, was common to the TRM5 and 3PR signatures. Proteins with differential abundance in the ACS training and test sets combined are in S7 Table and S2 Text.

Performance of the 3PR signature in the combined training plus test set was comparable to that of the TRM5 model (AUC 0.89, 95% CI 0.84–0.95) in samples between 1 and 180 days before TB diagnosis and in samples between 181 and 360 days before TB (AUC 0.72, 95% CI 0.64–0.81, Fig 3B). Notably, the 3PR signature also significantly discriminated between progressor and nonprogressor samples collected 361 to 720 days before TB, with an AUC of 0.71 (95% CI, 0.63–0.80). This enhanced performance at time points distal to TB may be due to a larger sample size of the discovery cohort used for the 3PR signature than that used for discovery of TRM5.

### Blind validation in an independent TB progressor and nonprogressor cohort

**Fig 4:**
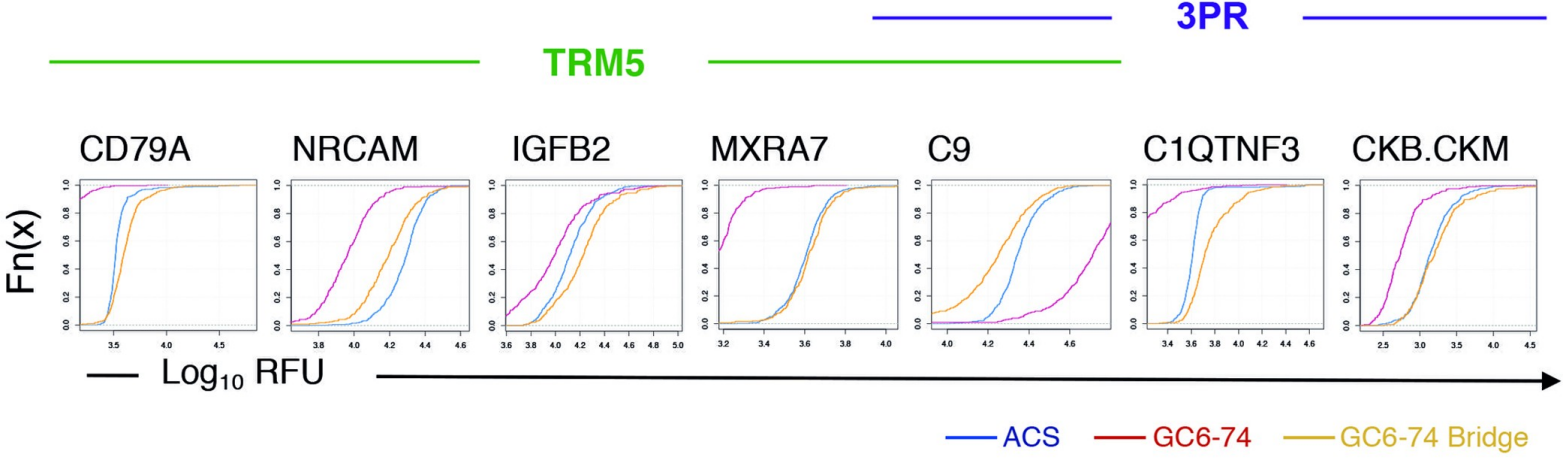
Cumulative distribution of select model proteins run on the custom validation slide arrays. Curves demonstrate a spectrum of distributions of RFU in the ACS versus the GC6–74 cohorts. Red curves represent GC6–74 samples prior to bridging, yellow curves represent GC6–74 postbridging, and blue curves represent ACS samples. ACS, Adolescent Cohort Study; GC6–74, Grand Challenges 6–74; RFU, raw fluoresence unit.

To validate the TRM5 and 3PR proteomic TB risk signatures in an independent cohort, we retrieved plasma samples from Gambian adult household contacts of TB cases who participated in the GC6–74 study [4,5] (S2 Table and S2 Text). Assignment of samples to progressor status, draw date, and participant were blinded. Raw fluorescence unit (RFU) signal levels in 45 ACS samples that were run on both the original SOMAscan discovery assay and the custom SOMAscan assay for bridging indicated a systematic intensity shift between signal levels. Despite the shift in mean signal intensity, most protein measurements generated with the ACS discovery array were well correlated with the original SOMAscan measurements, and the bulk intensity change was removed using the standard SOMAscan assay bridging procedure, which transforms the raw concentration ranges generated by the 45 ACS bridging samples on the validation array into the concentration ranges generated on the original discovery array. Fig 4 displays cumulative distribution functions of the TRM5 and 3PR analytes for the GC6–74 samples before and after assay bridging. A single progressor sample (of 61) and a single nonprogressor sample (of 193) failed SOMAscan operating procedure QC criteria—all other samples were deemed fit for analysis with the risk models. Distribution of progressor and nonprogressor signature scores in the ACS and GC6 cohorts was not different for the TRM5 model, although they were significantly different for the 3PR signature across discovery and validation assays (S4 Fig). Protein abundance data in the GC6 validation samples are in S6 Table and S2 Text.

**Fig 5:**
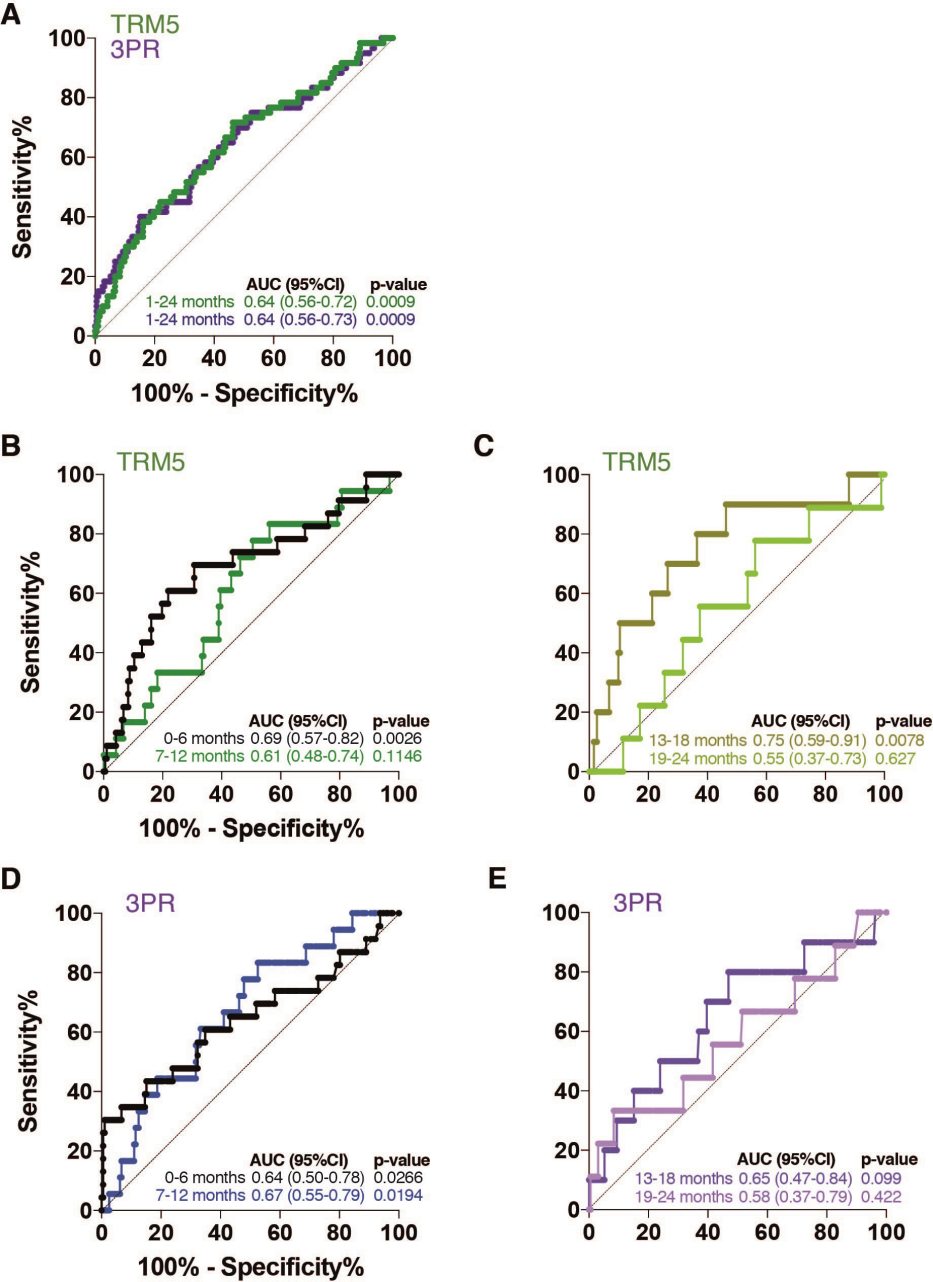
ROC-AUC analysis of the TRM5 and 3PR signatures for all GC6–74 validation set plasma samples, for (A) all prospectively collected samples, and (B–E) stratified by the time interval of each prospectively collected sample before TB diagnosis. 3PR, 3-protein pair-ratio; GC6–74, Grand Challenges 6–74; ROC-AUC, area under the receiver operator characteristic curve; TB, tuberculosis; TRM5, TB Risk Model 5.

Prognostic performance of both TRM5 and 3PR was determined on samples collected up to 2 years before the diagnosis of TB disease in the GC6–74 validation cohort (Fig 5). Both TRM5 and 3PR discriminated between Gambian progressors and nonprogressors within 1 year of TB diagnosis (TRM5: AUC 0.66 [95% CI 0.56–0.75]; 3PR: AUC 0.65 [0.55–0.75]).

Prognostic performance by both signatures was generally poor for samples collected from 1–2 years before diagnosis. When substratified into 6-month time windows before diagnosis of TB disease, performance of both models was, as anticipated, strongest most proximal to diagnosis (Table 2). The 3PR signature discriminated between progressor and nonprogressor samples collected 7–12 months before TB (AUC 0.67 [0.55–0.79], *P* = 0.019), and the TRM5 signature discriminated between progressor and nonprogressor samples 13–18 months before TB (AUC 0.75 [0.59–0.91], *P* = 0.0078). Neither signature showed significant performance for samples collected more than 18 months before TB diagnosis. After the bridge calibration procedure, only C9 and NrCAM were observed to have mean RFU values that were significantly different (Bonferroni *P* < 0.05) in the GC6–74 data set when explored in the ANOVA posthoc analysis for directionality of bias.

A target product profile for a test that predicts progression from TB infection to active disease, or an incipient TB test (ITT), was recently developed by FIND and WHO [21], which benchmarked the minimum sensitivity and specificity for such a test at ≥75% and ≥75%, respectively (optimal sensitivity and specificity were ≥90% and ≥90%). Neither TRM5 nor 3PR achieved these minimum criteria when tested for progression to incident TB diagnosed within a year of testing in the GC6 cohort; TRM5 achieved a sensitivity of 49% (95% CI 33%–65%) at a specificity of 75% (95% CI 68%–81%) and 3PR a sensitivity of 46% (95% CI 31%–63%) at a specificity of 75% (95% CI 68%–81%). By comparison, prognostic performance of CRP, the protein with the highest differential abundance between ACS progressors and nonprogressors, was promising in the combined ACS training and test sets in samples within 1 year of diagnosis (AUC 0.76; 95% CI 0.69–0.83) (S3A Fig). However, validation in the GC6–74 cohort was not statistically significant (AUC 0.62; 95% CI 0.49–0.74, *P* = 0.058). Despite this, CRP had a similar sensitivity of 41% (95 CI 22%–61%) at a specificity of 75% (95% CI 67%–82%) in the GC6–74 cohort (S3B and S3C Fig).

## Discussion

Using a well-characterized prospective longitudinal cohort of *M*. *tuberculosis*-infected South African adolescents, we discovered 2 prognostic protein signatures, TRM5 and 3PR, that successfully identified individuals at risk of incident TB disease risk within a year of the onset of disease symptoms. Validation of the prognostic performance of these signatures in an independent cohort of household contacts of TB patients from the Gambia represents a first step to an affordable and practical prognostic biomarker for TB.

While other proteomic biomarkers have been discovered with diagnostic potential for symptomatic TB disease [6–9], this outcome represents only one stage within the spectrum of *M*. *tuberculosis* infection. A biomarker with prognostic value that can identify asymptomatic individuals with incipient or subclinical disease would open the opportunity for early, targeted preventive treatment and the potential to curb *M*. *tuberculosis* transmission. A recent review of incipient or subclinical disease suggested that the number of individuals with these early stages of disease progression must be at least equivalent to the number of active TB cases: 10 million [22]. The only current tests that can identify those at risk of TB are interferon gamma release assays (IGRAs) or TSTs, which detect immunological sensitization to *M*. *tuberculosis*. These tests have low positive predictive value (PPV) for prognostic application [23,24], and the prevalence of TST+ or QFT+ people can be as high as 80% in countries endemic for TB. In fact, epidemiological models suggest that up to 23% of the global population may be infected with *M*. *tuberculosis* [25] and thus are at risk of disease progression, although a recent analysis has suggested that the proportion of individuals truly at risk of progression is likely smaller than the TST models suggest [26]. Regardless, these studies highlight the need for a prognostic test for incident TB that is more sensitive and specific than IGRAs and TSTs.

Neither TRM5 nor 3PR achieved the minimum criteria for an incipient TB test (ITT) set out by FIND and WHO [21], and it is clear that more work is needed to improve the performance of prognostic signatures based on proteins. The same was true of the prognostic performance of CRP. Notably, a recent diagnostic accuracy study conducted in 2 Ugandan HIV/AIDS clinics showed that point-of-care CRP screening of HIV-infected people with CD4 counts <351 cells per μL who were initiating antiretroviral therapy yielded 89% sensitivity and 72% specificity for culture confirmed TB [27]. The study supported use of CRP as a TB screening test to improve efficiency of case finding.

Nevertheless, our study reports, to the best of our knowledge, the first proteomic prognostic signature for TB and demonstrates feasibility of the approach. Prognostic transcriptomic signatures of TB risk have been developed using RNA sequencing [4,5], microarrays, in silico analysis of published data sets, as well as PCR-based methods [15,28]. While such transcriptomic signatures possess immense potential, their access to the market is hindered by high cost and the need to translate measurement of mRNA-based signatures to practical point-of-care devices for use in community healthcare or surveillance settings. A parsimonious proteomic signature could, in principal, be more amenable for adaptation to a portable and low-cost test, such as a lateral flow–based assay.

Interpretation of our results would benefit from verification with a different protein quantification technology, such as sandwich ELISA as proof-of-principle of antibody-based detection of proteins identified with SOMAmers, although commercial ELISA antibodies for detection of some of the proteins in the TRM5 and 3PR signatures at the appropriate biological range are limited. Ultimately, aptamer-based sandwich assays for analyte quantitation may be a viable alternative for point-of-care assays since aptamers can be manufactured reproducibly and do not require a cold chain. Translation to commercial methodologies would also allow easier uptake and external validation of these signatures in other populations and settings. This would also allow analysis of the effect on signature performance derived during the transition from the >3,000-plex SOMAscan discovery assay to the custom SOMAscan assay used for validation. We observed a systematic shift in signal magnitudes generated by the validation assay compared to the >3,000-plex discovery assay. Though the bridge calibration removed most of this artifact, there was still some residual shift in mean signal intensity for C9 and NrCAM, which may have contributed to the decrease in prognostic performance of TRM5 and 3PR in the GC6 validation cohort. Additionally, differences in disease epidemiology in the underlying populations, country of residence, strain of circulating *M*. *tuberculosis*, and/or the amount of heparin or other preanalytic processing variables in the plasma samples may also have contributed to a difference in performance between the ACS and GC6 cohorts. Regardless, our results showed that both proteomic signatures validated in the GC6 cohort and provide proof-of-principle that a prospective protein-based biomarker for incident TB is possible.

Our results of relative abundances of 2,872 plasma proteins in progressors and nonprogressors provide an opportunity to reflect on the biological pathways underlying progression from *M*. *tuberculosis* infection to active TB disease. We have previously shown that proteins associated with type I/II interferon responses (e.g., interferon gamma-inducible protein 10 [IP-10]) and complement cascade activation were elevated early during progression, up to 12 months before TB diagnosis, and are likely biomarkers of early incipient disease [10]. Elevated plasma proteins associated with myeloid inflammation, tissue repair, matrix remodeling, coagulation, and platelet activation were detected more proximal to TB diagnosis and suggestive of underlying pathology consistent with subclinical or active TB disease [10]. It was noteworthy that the methods employed to discover the TRM5 and 3PR signatures, which were completely agnostic to underlying biology, selected complement component C9 for inclusion in both proteomic signatures. This, along with the inclusion of C1qTNF3 in 3PR, further signifies the role of complement activation in TB disease progression, as shown by recent transcriptomic and proteomic studies [9–11]. C1qTNF3, which was less abundant in plasma from progressors than nonprogressors, has been shown to be inversely correlated with BMI and a proinflammatory obese state [29]. C1qTNF3 is a metabolic hormone with beneficial anti-inflammatory properties [30–32], and prior studies have found that obese individuals are at lower risk of incident TB [33] but greater risk of diabetes, which in itself is suggested as a TB risk factor [34]. The antidiabetes drug metformin, which has shown therapeutic potential in controling growth of *M*. *tuberculosis* [35], acts to increase C1qTNF3 levels [36]. Other studies have implicated low levels of C1qTNF3 in other inflammatory diseases such as rheumatoid arthritis [37], heart disease, lipid dysregulation, and apoptosis. Similarly, activation of the complement cascade in general and elevated C9 levels likely reflect the acute inflammatory responses and high type I interferon expression during TB disease progression [4,5,10,38]. The IGFBP-2 protein is implicated in growth and metabolism and was observed to increase during progressing infections [39], while plasma levels of insulin-like growth factor–binding proteins have been shown to change during TB treatment [40]. NrCAM is a member of the immunoglobulin superfamily and is important in cell adhesion and thought to be involved in immunity and pulmonary fibrosis [41,42]. While these inflammatory, immune activation, and tissue repair molecules provide some interpretation behind the biology of TB disease progression, the role of other differentially abundant proteins in the signatures, such as the dentin-associated ameloblastin (AMBN) and neuronal cell–associated NrCAM, are less clear and will require further investigation.

Our study had a number of limitations. Greater statistical power for signature discovery and validation would have been achieved with larger cohort sizes. It is critical that more progressor cohorts are assembled for future work on prognostic biomarkers for TB. In this light, the prospectively collected samples from the 76 progressors in both the ACS and GC6–74 cohorts—collected from 8,314 enrolled individuals—are of immense value. As such, the highly multiplexed SOMAscan assay was well suited for discovery, and the resulting data set is a valuable resource for the TB research community (S2 Text). The systematic shift in signal magnitudes generated by the validation assay compared to the discovery assay may be an important factor in the performance of TRM5 and 3PR in the validation cohort, as discussed above. New discovery using the entire ACS and GC6–74 data sets may allow discovery of a more universal signature, and it will be important to confirm the performance of these proteomic models on alternative platforms.

The performance of these signatures as diagnostic screening or triage tests should be further explored and compared with other protein-based diagnostic signatures [6–9], as such a signature with diagnostic utility would be an ideal tool for advancing the clinical care for TB. A next step is evaluation of the diagnostic performance in individuals with presumptive TB disease compared to those without confirmed TB but presenting with respiratory symptoms.

Successful validation of these proteomic signatures suggests that a simple proteomic test to predict progression to active TB disease is achievable. With further refinement and validation, the prospect of an affordable, point-of-care device to provide a tool to curb transmission is possible. While performance demonstrated here is not sufficient to meet minimal WHO guidelines for predicting progression of TB [1], the novelty of these prognostic signatures and the theoretical simplicity and robustness of a proteomic lateral flow test provides renewed hope in a prognostic marker for point-of-care.

## Supporting information

S1 Text The ACS and GC6–74 cohort study teams. ACS, Adolescent Cohort Study; GC6–74, Grand Challenges 6–74.
(DOCX)

S2 Text Supplementary text. (DOCX)

S1 Fig Schematic of the approach taken for discovery of the TRM5 and 3PR signatures. The TRM5 signature was discovered on a subset (the training set) of the ACS and then validated by blind prediction on the test set of the ACS. The 3PR signature was discovered on the full ACS set (training and test set combined). Both TRM5 and 3PR were validated by blind prediction on the GC6–74 cohort. 3PR, 3-protein pair-ratio; ACS, Adolescent Cohort Study; GC6–74, Grand Challenges 6–74; TRM5, TB Risk Model 5.
(TIF)

S2 Fig Q-Q plot displaying expected and observed KS *P* values for plasma protein abundances between progressors and nonprogressors. KS, Kolmogorov–Smirnov.
(TIF)

S3 Fig Receiver operator characteristic AUC analysis of CRP. (A) ACS training and test set progressor and nonprogressor plasma samples and GC6 validation set plasma samples from time points within 1 year of TB diagnosis. Sensitivity and specificity of CRP for the (B) ACS training and test set and (C) GC6 validation set. ACS, Adolescent Cohort Study; AUC, area under the curve; CRP, C-reactive protein; GC6, Grand Challenge 6; RFU, relative fluorescence units; TB, tuberculosis.
(TIF)

S4 Fig Distribution of signature scores for the TRM5 and 3PR in the combined ACS training and test cohorts and the GC6–74 validation cohort. Mann–Whitney test *P* values are shown for comparison of each signature on different progressor and nonprogressor samples run on the different SOMAscan assays. 3PR, 3-protein pair-ratio; ACS, Adolescent Cohort Study; GC6–74, Grand Challenges 6–74; SOMAscan; TRM5, TB Risk Model 5.
(TIF)

S1 Table Metadata of the ACS progressor and nonprogessor (control) cohort. ACS, Adolescent Cohort Study.
(XLSX)

S2 Table Metadata of the Gambian household contact progressor and nonprogressor (control) validation cohort. (XLSX)

S3 Table Annotation of proteins measured by SOMAscan. (XLSX)

S4 Table Raw abundances of proteins measured in samples from progressors and nonprogressors from the ACS. ACS, Adolescent Cohort Study.
(XLSX)

S5 Table Differential protein abundances between progressors and nonprogressors in the training set from the ACS, ranked by ascending bhFDR. ACS, Adolescent Cohort Study; bhFDR, Benjamini–Hochberg False Discovery Rate.
(XLSX)

S6 Table Raw abundances of 150 proteins measured in the GC6–74 Gambian validation cohort. GC6–74, Grand Challenges 6–74.
(XLSX)

S7 Table Differential protein abundances between progressors and nonprogressors in the training and test sets (combined) from the ACS, ranked by ascending bhFDR. ACS, Adolescent Cohort Study; bhFDR, Benjamini–Hochberg False Discovery Rate.
(XLSX)

S1. TRIPOD checklist TRIPOD, transparent reporting of a multivariable prediction model for individual prognosis or diagnosis. (DOCX)

## Abbreviations

ACS: Adolescent Cohort Study
AMBN: ameloblastin
AUC: area under the curve
BH: Benjamini–Hochberg
bhFDR: Benjamini–Hochberg False Discovery Rate
CD79A: B-cell antigen receptor complex–associated protein
CK-MB: Creatine Kinase type M/type B
CRP: C-reactive protein
GALT-1: Galactose-1-phosphate uridyl transferase 1
GC6–74: Grand Challenges 6–74
IGFBP-2: insulin-like growth factor-binding protein 2
IGRA: interferon gamma release assay
IP-10: interferon gamma-inducible protein 10
ITT: incipient TB test
MMP-1: Matrix Metalloproteinase 1
MXRA-7: Matrix-Remodeling Associated 7 protein
NrCAM: neuronal cell-adhesion molecule
N/A: not available
PPV: positive predictive value
SOMAmer: slow off-rate modified DNA aptamer
TB: tuberculosis
TRM5: TB Risk Model 5
TST: tuberculin skin test
3PR: 3-protein pair-ratio

## References

[1] World Health Organization. GLOBAL TUBERCULOSIS REPORT 2018. 2018;: 1–243.

[2] World Health Organization. Global tuberculosis report 2015. 2015.

[3] Ottenhoff THM, Ellner JJ, Kaufmann SHE. Ten challenges for TB biomarkers. Tuberculosis (Edinb). 2012;92 Suppl 1: S17–20. 10.1016/S1472-9792(12)70007-0 22441153

[4] Zak DE, Penn-Nicholson A, Scriba TJ, Thompson E, Suliman S, Amon LM, et al A blood RNA signature for tuberculosis disease risk: a prospective cohort study. Lancet. 2016;387: 2312–2322. 10.1016/S0140-6736(15)01316-1 27017310 PMC5392204

[5] Suliman S, Thompson E, Sutherland J, Weiner Rd J, Ota MOC, Shankar S, et al Four-gene Pan-African Blood Signature Predicts Progression to Tuberculosis. Am J Respir Crit Care Med. 2018;197: 1198–1208. 10.1164/rccm.201711-2340OC 29624071 PMC6019933

[6] Yoon C, Chaisson LH, Patel SM, Allen IE, Drain PK, Wilson D, et al Diagnostic accuracy of C-reactive protein for active pulmonary tuberculosis: a meta-analysis. Int J Tuberc Lung Dis. 2017;21: 1013–1019. 10.5588/ijtld.17.0078 28826451 PMC5633000

[7] Chegou NN, Sutherland JS, Malherbe S, Crampin AC, Corstjens PLAM, Geluk A, et al Diagnostic performance of a seven-marker serum protein biosignature for the diagnosis of active TB disease in African primary healthcare clinic attendees with signs and symptoms suggestive of TB. Thorax. 2016;: thoraxjnl–2015–207999. 10.1136/thoraxjnl-2015-207999 27146200

[8] De Groote MA, Nahid P, Jarlsberg L, Johnson JL, Weiner M, Muzanyi G, et al Elucidating novel serum biomarkers associated with pulmonary tuberculosis treatment. PLoS ONE. 2013;8: e61002 10.1371/journal.pone.0061002 23637781 PMC3630118

[9] De Groote MA, Sterling DG, Hraha T, Russell TM, Green LS, Wall K, et al Discovery and Validation of a Six-Marker Serum Protein Signature for the Diagnosis of Active Pulmonary Tuberculosis. Land GA, editor. J Clin Microbiol. 2017;55: 3057–3071. 10.1128/JCM.00467-17 28794177 PMC5625392

[10] Scriba TJ, Penn-Nicholson A, Shankar S, Hraha T, Thompson EG, Sterling D, et al Sequential inflammatory processes define human progression from M. tuberculosis infection to tuberculosis disease. Sassetti CM, editor. PLoS Pathog. 2017;13: e1006687 10.1371/journal.ppat.1006687 29145483 PMC5689825

[11] Esmail H, Lai RP, Lesosky M, Wilkinson KA, Graham CM, Horswell S, et al Complement pathway gene activation and rising circulating immune complexes characterize early disease in HIV-associated tuberculosis. Proc Natl Acad Sci USA. 2018;115: E964–E973. 10.1073/pnas.1711853115 29339504 PMC5798330

[12] Mahomed H, Hawkridge T, Verver S, Geiter L, Hatherill M, Abrahams DA, et al Predictive factors for latent tuberculosis infection among adolescents in a high-burden area in South Africa. Int J Tuberc Lung Dis. 2011;15: 331–336. 21333099

[13] Mahomed H, Ehrlich R, Hawkridge T, Hatherill M, Geiter L, Kafaar F, et al TB Incidence in an Adolescent Cohort in South Africa. PLoS ONE; 2013;8: e59652 10.1371/journal.pone.0059652 23533639 PMC3606161

[14] Health SADO. National Tuberculosis Management Guidelines 2014. South African Family Practice. 2014 pp. 3–4. 10.1080/20786204.2007.10873647

[15] Duffy FJ, Thompson E, Downing K, Suliman S, Mayanja-Kizza H, Boom WH, et al A Serum Circulating miRNA Signature for Short-Term Risk of Progression to Active Tuberculosis Among Household Contacts. Front Immunol. 2018;9: 661 10.3389/fimmu.2018.00661 29706954 PMC5908968

[16] Gold L, Ayers D, Bertino J, Bock C, Bock A, Brody EN, et al Aptamer-based multiplexed proteomic technology for biomarker discovery. PLoS ONE. 2010;5: e15004 10.1371/journal.pone.0015004 21165148 PMC3000457

[17] Ganz P, Heidecker B, Hveem K, Jonasson C, Kato S, Segal MR, et al Development and Validation of a Protein-Based Risk Score for Cardiovascular Outcomes Among Patients With Stable Coronary Heart Disease. JAMA. 2016;315: 2532–2541. 10.1001/jama.2016.5951 27327800

[18] Mack GA, Wolfe DA. K-Sample Rank Tests for Umbrella Alternatives. J Am Stat Ass. Taylor & Francis; 1981;76: 175–181. 10.1080/01621459.1981.10477625

[19] Thompson EG, Du Y, Malherbe ST, Shankar S, Braun J, Valvo J, et al Host blood RNA signatures predict the outcome of tuberculosis treatment. Tuberculosis (Edinb). 2017;107: 48–58. 10.1016/j.tube.2017.08.004 29050771 PMC5658513

[20] Thompson EG, Shankar S, Gideon HP, Braun J, Valvo J, Skinner JA, et al Prospective Discrimination of Controllers From Progressors Early After Low-Dose Mycobacterium tuberculosis Infection of Cynomolgus Macaques using Blood RNA Signatures. J Infect Dis. 2018;217: 1318–1322. 10.1093/infdis/jiy006 29325117 PMC6018950

[21] World Health Organization. Consensus meeting report: development of a target product profile (TPP) and a framework for evaluation for a test for predicting progression from tuberculosis Geneva: 2017 (WHO/HTM/TB/2017.18).

[22] Drain PK, Bajema KL, Dowdy D, Dheda K, Naidoo K, Schumacher SG, et al Incipient and Subclinical Tuberculosis: a Clinical Review of Early Stages and Progression of Infection. Clin Microbiol Rev. 2018;31 10.1128/CMR.00021-18 30021818 PMC6148193

[23] Diel R, Loddenkemper R, Nienhaus A. Predictive value of interferon-γ release assays and tuberculin skin testing for progression from latent TB infection to disease state: a meta-analysis. Chest. 2012;142: 63–75. 10.1378/chest.11-3157 22490872

[24] Rangaka MX, Wilkinson KA, Glynn JR, Ling D, Menzies D, Mwansa-Kambafwile J, et al Predictive value of interferon-γ release assays for incident active tuberculosis: a systematic review and meta-analysis. Lancet Infect Dis. 2012;12: 45–55. 10.1016/S1473-3099(11)70210-9 21846592 PMC3568693

[25] Houben RMGJ, Dodd PJ. The Global Burden of Latent Tuberculosis Infection: A Re-estimation Using Mathematical Modelling. Metcalfe JZ, editor. PLoS Med. 2016;13: e1002152 10.1371/journal.pmed.1002152 27780211 PMC5079585

[26] Behr MA, Edelstein PH, Ramakrishnan L. Revisiting the timetable of tuberculosis. BMJ. 2018;362: k2738 10.1136/bmj.k2738 30139910 PMC6105930

[27] Yoon C, Semitala FC, Atuhumuza E, Katende J, Mwebe S, Asege L, et al Point-of-care C-reactive protein-based tuberculosis screening for people living with HIV: a diagnostic accuracy study. Lancet Infect Dis. 2017;17: 1285–1292. 10.1016/S1473-3099(17)30488-7 28847636 PMC5705273

[28] Sweeney TE, Braviak L, Tato CM, Khatri P. Genome-wide expression for diagnosis of pulmonary tuberculosis: a multicohort analysis. Lancet Respir Med. 2016;4: 213–224. 10.1016/S2213-2600(16)00048-5 26907218 PMC4838193

[29] Wolf RM, Steele KE, Peterson LA, Magnuson TH, Schweitzer MA, Wong GW. Lower Circulating C1q/TNF-Related Protein-3 (CTRP3) Levels Are Associated with Obesity: A Cross-Sectional Study. Zhang Y, editor. PLoS ONE. 2015;10: e0133955–11. 10.1371/journal.pone.0133955 26222183 PMC4519328

[30] Weigert J, Neumeier M, Schäffler A, Fleck M, Schölmerich J, Schütz C, et al The adiponectin paralog CORS-26 has anti-inflammatory properties and is produced by human monocytic cells. FEBS Lett. 2005;579: 5565–5570. 10.1016/j.febslet.2005.09.022 16213490

[31] Kopp A, Bala M, Buechler C, Falk W, Gross P, Neumeier M, et al C1q/TNF-Related Protein-3 Represents a Novel and Endogenous Lipopolysaccharide Antagonist of the Adipose Tissue. Endocrinol. 2010;151: 5267–5278. 10.1210/en.2010-0571 20739398

[32] Schmid A, Kopp A, Hanses F, Karrasch T, Schäffler A. C1q/TNF-related protein-3 (CTRP-3) attenuates lipopolysaccharide (LPS)-induced systemic inflammation and adipose tissue Erk-1/-2 phosphorylation in mice in vivo. Biochem Biophys Res Commun; 2014;452: 8–13. 10.1016/j.bbrc.2014.06.054 24996172

[33] Lin H-H, Wu C-Y, Wang C-H, Fu H, Lönnroth K, Chang Y-C, et al Association of Obesity, Diabetes, and Risk of Tuberculosis: Two Population-Based Cohorts. Clin Infect Dis. 2017;66: 699–705. 10.1093/cid/cix852 29029077 PMC5850624

[34] Leung CC, Lam TH, Chan WM, Yew WW, Ho KS, Leung G, et al Lower risk of tuberculosis in obesity. Arch Intern Med. AMA; 2007;167: 1297–1304. 10.1001/archinte.167.12.1297 17592104

[35] Singhal A, Jie L, Kumar P, Hong GS, Leow MKS, Paleja B, et al Metformin as adjunct antituberculosis therapy. Sci Transl Med. 2014;6: 263ra159–263ra159. 10.1126/scitranslmed.3009885 25411472

[36] Tan BK, Chen J, Hu J, Amar O, Mattu HS, Adya R, et al Metformin increases the novel adipokine cartonectin/CTRP3 in women with polycystic ovary syndrome. J Clin Endocrinol Metab. 2013;98: E1891–900. 10.1210/jc.2013-2227 24152681

[37] Murayama MA, Kakuta S, Maruhashi T, Shimizu K, Seno A, Kubo S, et al CTRP3 plays an important role in the development of collagen-induced arthritis in mice. Biochem Biophys Res Commun. 2014;443: 42–48. 10.1016/j.bbrc.2013.11.040 24269820

[38] Berry MPR, Graham CM, McNab FW, Xu Z, Bloch SAA, Oni T, et al An interferon-inducible neutrophil-driven blood transcriptional signature in human tuberculosis. Nature. 2010;466: 973–977. 10.1038/nature09247 20725040 PMC3492754

[39] Helle SI, Ueland T, Ekse D, Frøland SS, Holly JM, Lønning PE, et al The insulin-like growth factor system in human immunodeficiency virus infection: relations to immunological parameters, disease progression, and antiretroviral therapy. J Clin Endocrinol Metab. 2001;86: 227–233. 10.1210/jcem.86.1.7135 11232005

[40] Nahid P, Bliven-Sizemore E, Jarlsberg LG, De Groote MA, Johnson JL, Muzanyi G, et al Aptamer-based proteomic signature of intensive phase treatment response in pulmonary tuberculosis. Tuberculosis (Edinb). 2014;94: 187–196. 10.1016/j.tube.2014.01.006 24629635 PMC4028389

[41] Katoh M. Multi‑layered prevention and treatment of chronic inflammation, organ fibrosis and cancer associated with canonical WNT/β‑catenin signaling activation (Review). Int J Mol Med. 2018;42: 713–725. 10.3892/ijmm.2018.3689 29786110 PMC6034925

[42] Volkmer H, Schreiber J, Rathjen FG. Regulation of adhesion by flexible ectodomains of IgCAMs. Neurochem Res. 2013;38: 1092–1099. 10.1007/s11064-012-0888-9 23054071

