# Correction: Discovery and validation of a prognostic proteomic signature for tuberculosis progression: A prospective cohort study

**DOI:** 10.1371/journal.pmed.1002880

**Published:** 2019-07-18

**Authors:** Adam Penn-Nicholson, Thomas Hraha, Ethan G. Thompson, David Sterling, Stanley Kimbung Mbandi, Kirsten M. Wall, Michelle Fisher, Sara Suliman, Smitha Shankar, Willem A. Hanekom, Nebojsa Janjic, Mark Hatherill, Stefan H. E. Kaufmann, Jayne Sutherland, Gerhard Walzl, Mary Ann De Groote, Urs Ochsner, Daniel E. Zak, Thomas J. Scriba, ACS and GC6–74 cohort study groups

In supplemental file S4 Fig, the labels for “controls” and “progressors” were switched. The corrected S4 Fig can be found here.

## Supporting information

S4 Fig Distribution of signature scores for the TRM5 and 3PR in the combined ACS training and test cohorts and the GC6–74 validation cohort. Mann–Whitney test P values are shown for comparison of each signature on different progressor and nonprogressor samples run on the different SOMAscan assays. 3PR, 3-protein pair-ratio; ACS, Adolescent Cohort Study; GC6–74, Grand Challenges 6–74; SOMAscan; TRM5, TB Risk Model 5.
(TIF)

## References

[1] Penn-Nicholson A, Hraha T, Thompson EG, Sterling D, Mbandi SK, Wall KM, et al (2019) Discovery and validation of a prognostic proteomic signature for tuberculosis progression: A prospective cohort study. PLoS Med 16(4): e1002781 10.1371/journal.pmed.1002781 30990820 PMC6467365

