# Supplementary material for: Discovery and validation of a prognostic proteomic signature for tuberculosis progression: A prospective cohort study
Source: PLoS Med. 2019 Apr 16;16(4):e1002781. doi: 10.1371/journal.pmed.1002781 (PMC6467365; doi:10.1371/journal.pmed.1002781)
Supplement: S1 Text — ACS, Adolescent Cohort Study; GC6–74, Grand Challenges 6–74. (DOCX) [file pmed.1002781.s001.docx]

**The GC6-74 cohort study team:**

Stellenbosch University, South Africa:

Gerhard Walzl, Gillian F. Black, Gian van der Spuy, Kim Stanley,

Magdalena Kriel, Nelita Du Plessis, Nonhlanhla Nene, Teri Roberts,

Leanie Kleynhans, Andrea Gutschmidt, Bronwyn Smith, Nonhlanhla Nene,

Andre G. Loxton, Novel N. Chegou, Gerhardus Tromp, David Tabb

Department of Infectious Diseases, Leiden University Medical Centre,

Leiden, The Netherlands:

Tom H.M. Ottenhoff, Michel R. Klein, Marielle C. Haks, Kees L.M.C.

Franken, Annemieke Geluk, Krista E van Meijgaarden, Simone A Joosten

Tuberculosis Research Unit, Department of Medicine, Case Western

Reserve University School of Medicine and University Hospitals Case

Medical Center, Cleveland, Ohio, USA:

W. Henry Boom, Bonnie Thiel

Department of Medicine and Department of Microbiology, College of

Health Sciences, Faculty of Medicine, Makerere University, Kampala,

Uganda:

Harriet Mayanja-Kizza, Moses Joloba, Sarah Zalwango, Mary Nsereko,

Brenda Okwera, Hussein Kisingo

Department of Immunology, Max Planck Institute for Infection Biology,

Berlin, Germany:

Stefan H.E. Kaufmann (GC6-74 Principal Investigator), Shreemanta K.

Parida, Robert Golinski, Jeroen Maertzdorf, January Weiner 3rd, Marc

Jacobson

Department of Immunology and Infection, Faculty of Infectious and

Tropical Diseases, London School of Hygiene & Tropical Medicine,

London, United Kingdom:

Hazel Dockrell, Steven Smith, Patricia Gorak-Stolinska, Yun-Gyoung Hur,

Maeve Lalor, Ji-Sook Lee

Karonga Prevention Study, Chilumba, Malawi:

Amelia C Crampin, Neil French, Bagrey Ngwira, Anne Ben-Smith, Kate

Watkins, Lyn Ambrose, Felanji Simukonda, Hazzie Mvula, Femia

Chilongo, Jacky Saul, Keith Branson

South African Tuberculosis Vaccine Initiative, Institute of Infectious

Disease and Molecular Medicine and Division of Immunology, Department of

Pathology, University of Cape Town, Cape Town, South Africa:

Sara Suliman, Thomas J. Scriba, Hassan Mahomed, E. Jane Hughes,

Nicole Bilek, Mzwandile Erasmus, Onke Xasa, Ashley Veldsman, Katrina

Downing, Michelle Fisher, Adam Penn-Nicholson, Humphrey Mulenga,

Brian Abel, Mark Bowmaker, Benjamin Kagina, William Kwong Chung,

Willem A. Hanekom

Aeras, Rockville, MD, USA:

Jerry Sadoff, Donata Sizemore, S Ramachandran, Lew Barker, Michael

Brennan, Frank Weichold, Stefanie Muller, Larry Geiter

Ethiopian Health & Nutrition Research Institute, Addis Ababa, Ethiopia:

Desta Kassa, Almaz Abebe, Tsehayenesh Mesele, Belete Tegbaru

University Medical Centre, Utrecht, The Netherlands:

Debbie van Baarle, Frank Miedema

Armauer Hansen Research Institute, Addis Ababa, Ethiopia:

Rawleigh Howe, Adane Mihret, Abraham Aseffa, Yonas Bekele, Rachel

Iwnetu, Mesfin Tafesse, Lawrence Yamuah

Vaccines & Immunity Theme, Medical Research Council Unit, Fajara, The

Gambia:

Martin Ota, Jayne Sutherland, Philip Hill, Richard Adegbola, Tumani

Corrah, Martin Antonio, Toyin Togun, Ifedayo Adetifa, Simon Donkor

Department of Infectious Disease Immunology, Statens Serum Institute,

Copenhagen, Denmark:

Peter Andersen, Ida Rosenkrands, Mark Doherty, Karin Weldingh

Department of Microbiology and Immunology, Stanford University,

Stanford, California, USA:

Gary Schoolnik, Gregory Dolganov, Tran Van

**The ACS cohort study team:**

South African Tuberculosis Vaccine Initiative, Institute of Infectious

Disease and Molecular Medicine and Division of Immunology, Department of

Pathology, University of Cape Town, Cape Town, South Africa:

Fazlin Kafaar, Leslie Workman, Humphrey Mulenga, Thomas J. Scriba, E.

Jane Hughes, Nicole Bilek, Mzwandile Erasmus, Onke Xasa, Ashley

Veldsman, Yolundi Cloete, Deborah Abrahams, Sizulu Moyo, Sebastian

Gelderbloem, Michele Tameris, Hennie Geldenhuys, Willem Hanekom,

School of Public Health and Family Medicine, University of Cape Town,

Cape Town, South Africa:

Rodney Ehrlich

KNCV Tuberculosis Foundation, The Hague, and Amsterdam Institute of

Global Health and Development, Academic Medical Centre, Amsterdam,

The Netherlands:

Suzanne Verver

Aeras, Rockville, MD, USA:

Larry Geiter
