# Supplementary material for: Discovery and validation of a prognostic proteomic signature for tuberculosis progression: A prospective cohort study
Source: PLoS Med. 2019 Apr 16;16(4):e1002781. doi: 10.1371/journal.pmed.1002781 (PMC6467365; doi:10.1371/journal.pmed.1002781)
Supplement: S2 Text — (DOCX) [file pmed.1002781.s002.docx]

**Supplementary Text**

Discovery and Validation of a Prognostic Proteomic Signature for Tuberculosis Progression: a prospective cohort study

Adam Penn-Nicholson et al.,

**Detailed definition of progressors and non-progressors in the ACS and in GC6-74 cohorts**

**Discovery cohort**

The Adolescent Cohort Study (ACS) determined the prevalence and incidence of *M. tuberculosis* infection and disease among adolescents from the Cape Town region of South Africa. Selection of progressors and non-progressors for discovery and verification of proteomic signatures, adolescents with latent *M. tuberculosis* infection at enrollment were eligible; *M. tuberculosis* infection was diagnosed by a positive QuantiFERON TB GOLD In-Tube Assay (QFT, Quantiferon; >0.35 IU/mL) and/or a positive tuberculin skin test (TST, 0.1mL dose of Purified Protein Derivative RT-23, 2-TU, Staten Serum Institute; >10mm).

Adolescents who developed active tuberculosis disease during 2 years of follow-up were included as “progressors”. Participants that were either exposed to tuberculosis patients, or had symptoms suggestive of tuberculosis, were evaluated clinically and by sputum smear, culture and chest roentgenography. Tuberculosis was defined as intrathoracic disease, with either two sputum smears positive for acid-fast bacilli or one positive sputum culture confirmed as *M. tuberculosis* complex (Mycobacterial growth indicator tube, BD BioSciences). Participants who developed tuberculosis within 6 months of enrolment were excluded on the basis that they may represent individuals with active but as yet asymptomatic tuberculosis disease.

Five ACS participants who were not infected with *M. tuberculosis* at enrollment but who converted to a positive QFT and/or TST, and ultimately developed tuberculosis disease at least 6 months post QFT/TST conversion, were also included as progressors. A subset of ACS participants from the parent study, who had a negative QFT at baseline, were followed for incident tuberculosis for up to 3 years after the last QFT (5 years in total) through biannual study visits and passive surveillance of health facility records. The follow-up of these participants was longer than the 2 years applying to most participants.

All ACS patients with tuberculosis disease were offered a HIV test; HIV infected patients were excluded from the progressor/non-progressor study. HIV testing of healthy adolescents who participated in the study was not permitted by the Human Research Ethics Committee of the University of Cape Town; this committee also did not allow post-hoc, anonymous HIV testing. Regardless, the HIV incidence rate in adolescents diagnosed with active tuberculosis was <2% (1 out of 61 who were offered and accepted testing), and since HIV is a risk factor for tuberculosis, we expect the HIV prevalence among healthy adolescents (from whom controls were identified) to be very low.

For each ACS progressor, two matched non-progressors were identified. Non-progressors were selected from ACS participants who remained healthy during follow-up, and were matched to progressors by age at enrolment, gender, ethnicity, school of attendance, and presence or absence of prior episodes of tuberculosis disease.

**Validation Cohort**

Among GC6-74 participants from The Gambia, progressors were defined as having intrathoracic tuberculosis by one of three categories: First, two positive sputum cultures (MGIT); second, one positive sputum culture and/or a positive sputum smear, and clinical signs and symptoms compatible with tuberculosis and/or a chest roentgenogram compatible with active pulmonary tuberculosis; third, two positive sputum smears with clinical signs and symptoms compatible with tuberculosis or a chest roentgenogram compatible with active pulmonary tuberculosis. Progressors were excluded if they developed disease within 90 days of enrollment, for reasons mentioned above. Controls were matched to progressors based on age category (<18, 18-25, 26-35, ≥36 years of age), gender and year of enrollment.

**Analysis strategy for signature discovery, verification and validation**

A prospective analysis plan was in place before any analysis of the proteomic data, as is standard operating procedure at SomaLogic. Components of this plan included analysis of sample quality, data transformation to stabilize variance. The plan predetermined the nominal significance level of 5% for assessing differential expression with false discovery rate to account for multiple testing using Storey's method. Statistical modeling was planned on longitudinal ACS samples from a training sub-cohort of progressors and non-progressors that was pre-selected and agreed upon by investigators at UCT, CIDR and Somalogic, to optimize the TRM5 signature and a hold-out, training sub-cohort (verification samples) of progressors and non-progressors to assess performance of the final model with empirical (bootstrap) confidence intervals. A schematic of the approach taken for discovery, verification and blind validation is shown in Supplementary Figure 1. The verification samples were blinded and separated from the discovery/training set by UCT investigators prior to shipment, and were planned to be stored at SomaLogic while the discovery set was assayed and the classifiers were constructed. A precise specification of the TRM5 model suitable for computation, as well as the estimated performance on the “hold out” test set (33% of the ACS cohort) was planned to be shared with UCT and CIDR collaborators prior to assaying the verification set. SomaLogic remained blinded to the diagnostic label (TB, non-TB and time points) during assay of the verification samples. Diagnostic labels were planned to be revealed by the UCT collaborators until predictions were recorded on the verification samples and disclosed to the whole group.

Once the TRM5 signature had been discovered and verified on the ACS progressor and non-progressor cohort, the pair-ratio signature, 3PR, was discovered on the entire ACS progressor and non-progressor cohort. Fitting this pair-ratio model on the full ACS progressor and non-progressor data was not part of the prospective analysis plan. It was performed as a post-hoc analysis in light of the finding that the TRM5 signature did not significantly discriminate between progressor and non-progressor samples collected more than 1 year before TB diagnosis (Figure 2) and was informed by the work on transcriptomic signatures, which showed that a 16-gene mRNA signature allowed significant discrimination between samples from progressors and non-progressors at time points more than 12 months before TB diagnosis.

After discovery of the 3PR signature, the GC6-74 validation samples were assayed at Somalogic. These samples were re-coded at UCT before shipment to Somalogic, such that all investigators at Somalogic, CIDR and those at UCT who would work on signature predictions were blinded to progressor and non-progressor status as well as the time point of the samples relative to TB diagnosis. The TRM5 and 3PR signature R codes were shared and predictions on the validation samples were made simultaneously at Somalogic, UCT and CIDR, and per-sample results were compared to confirm that predictions were reproducible. Once all investigators had agreed that predictions for both signatures were consistent, the samples were unblinded and performance of the signatures was assessed.

**Detailed methodology relating to TRM5 signature discovery**

To discover the TRM5 signature, the non-parametric Kolmogorov-Smirnoff (KS) test was used to identify proteins differentially expressed between progressors and non-progressors. A QQ plot on the KS p-values (S2 Fig) indicated that the p-values for the most significant proteins deviate from the expected null distribution, suggesting that several plasma proteins exhibit abundances that differ significantly between progressors and non-progressors under very stringent control for multiple testing. We find 20 proteins at a 1% significance level after stringent Bonferonni correction, which controls for the Family-Wise Error Rate (as opposed to the False Discovery Rate). In addition we sought “responsive proteins”, those with differential temporal response across time, using the nonparametric Mack-Wolfe [1] test with discrete 6-month bins, was used to identify proteins with time-varying expression levels in either progressors or non-progressors. This is a distribution-free test for the restricted class of “umbrella alternative” hypotheses that posit a monotonic mean response on either side of the group exhibiting the extreme response. This class of alternative hypotheses covers both monontonic mean responses and responses that achieve a minimum or maximum at one of the interior groups. Differentially expressed proteins were identified using a 5% Benjamini-Hochberg (BH) corrected false discovery rate.

The 5-protein TB risk model (TRM5) is a Mahalanobis distance classifier. Screening proteins with the KS and Mack-Wolfe tests produced a reduced list of 21 candidate proteins that both distinguished progressors from non-progressors and exhibited non-trivial differential response over time. Our goal was to develop a model that could be deployed on an inexpensive assay platform in the field so we considered models with at most 5 proteins. We examined all 27,895 possible combinations of up to 5 proteins chosen from the reduced list of candidates. For each combination of proteins, the Mahalanobis model mean and robust covariance estimates were generated – the latter using the minimum covariance determinant (MCD) to reduce the influence of outliers. The TRM5 model functions as an anomaly detector leveraging the joint distribution of model proteins in the “non-progressor” class to provide an estimate of the probability of disease progression. The script for computing the TRM5 signature is available from SomaLogic upon request.

**Detailed methodology relating to 3PR signature discovery**

The 3 protein model of risk for TB disease progression was developed by applying the Pair Ratios algorithm to the ACS progressor and non-progressor cohort from the combined training and test sets, in a variation on the pairwise approach used to discover the 16-gene ACS COR and the RISK4 signatures [2-5].  The Pair Ratios algorithm results in an ensemble of protein pairs which each provide a risk score for each sample. The final model score for each sample is then computed as the average over the scores generated from each pair. The pairs in the signature were selected via a two step process. First, a univariate feature selection was applied to identify the proteins that are individually highest and lowest in progressors relative to non-progressors. The number of proteins selected during the univariate selection step is a hyperparameter that was optimized via leave-one-out cross-validation. Using the pool of proteins selected during the univariate selection step, all pairs involving one protein higher in the progressors and one protein higher in the non-progressors were assembled. For each pair of proteins, the log10 transformed fold-change in raw concentration was computed, where the directionality of the fold-change was chosen such that progressors had on average higher values. A score table was then constructed for each pair, such that the score for each ratio is equal to 1/2 times the fraction of training set progressors with a smaller ratio plus 1/2 times the fraction of training set non-progressors with a smaller ratio. Scores therefore range between 0 and 1, with scores closer to 1 indicating that a ratio is indicative of progression.  The final 3 protein signature was selected based on a balance between signature size and performance. Out of all three-protein signatures, the 3PR signature optimally stratified the training set, and 3PR performance did not statistically significantly differ from the optimal larger signatures. Because the 3PR signature is ratiometric and involves only three proteins, it is ideally suited for translation to a targeted platform. The script for computing for the 3PR signature is available at BitBucket (https://bitbucket.org/satvi/3pr).

**Supplementary References**

1. Mack GA, Wolfe DA. K-Sample Rank Tests for Umbrella Alternatives. Journal of the American Statistical Association. Taylor & Francis; 1981;76: 175–181. doi:10.1080/01621459.1981.10477625

2. Zak DE, Penn-Nicholson A, Scriba TJ, Thompson E, Suliman S, Amon LM, et al. A blood RNA signature for tuberculosis disease risk: a prospective cohort study. Lancet. 2016;387: 2312–2322. doi:10.1016/S0140-6736(15)01316-1

3. Suliman S, Thompson E, Sutherland J, Weiner Rd J, Ota MOC, Shankar S, et al. Four-gene Pan-African Blood Signature Predicts Progression to Tuberculosis. Am J Respir Crit Care Med. 2018;197: 1198–1208. doi:10.1164/rccm.201711-2340OC

4. Thompson EG, Du Y, Malherbe ST, Shankar S, Braun J, Valvo J, et al. Host blood RNA signatures predict the outcome of tuberculosis treatment. Tuberculosis (Edinb). 2017;107: 48–58. doi:10.1016/j.tube.2017.08.004

5. Thompson EG, Shankar S, Gideon HP, Braun J, Valvo J, Skinner JA, et al. Prospective Discrimination of Controllers From Progressors Early After Low-Dose Mycobacterium tuberculosis Infection of Cynomolgus Macaques using Blood RNA Signatures. Journal of Infectious Diseases. 2018;217: 1318–1322. doi:10.1093/infdis/jiy006
