# Supplementary figures and images for: Discovery and validation of a prognostic proteomic signature for tuberculosis progression: A prospective cohort study
Source: PLoS Med. 2019 Apr 16;16(4):e1002781. doi: 10.1371/journal.pmed.1002781 (PMC6467365; doi:10.1371/journal.pmed.1002781)

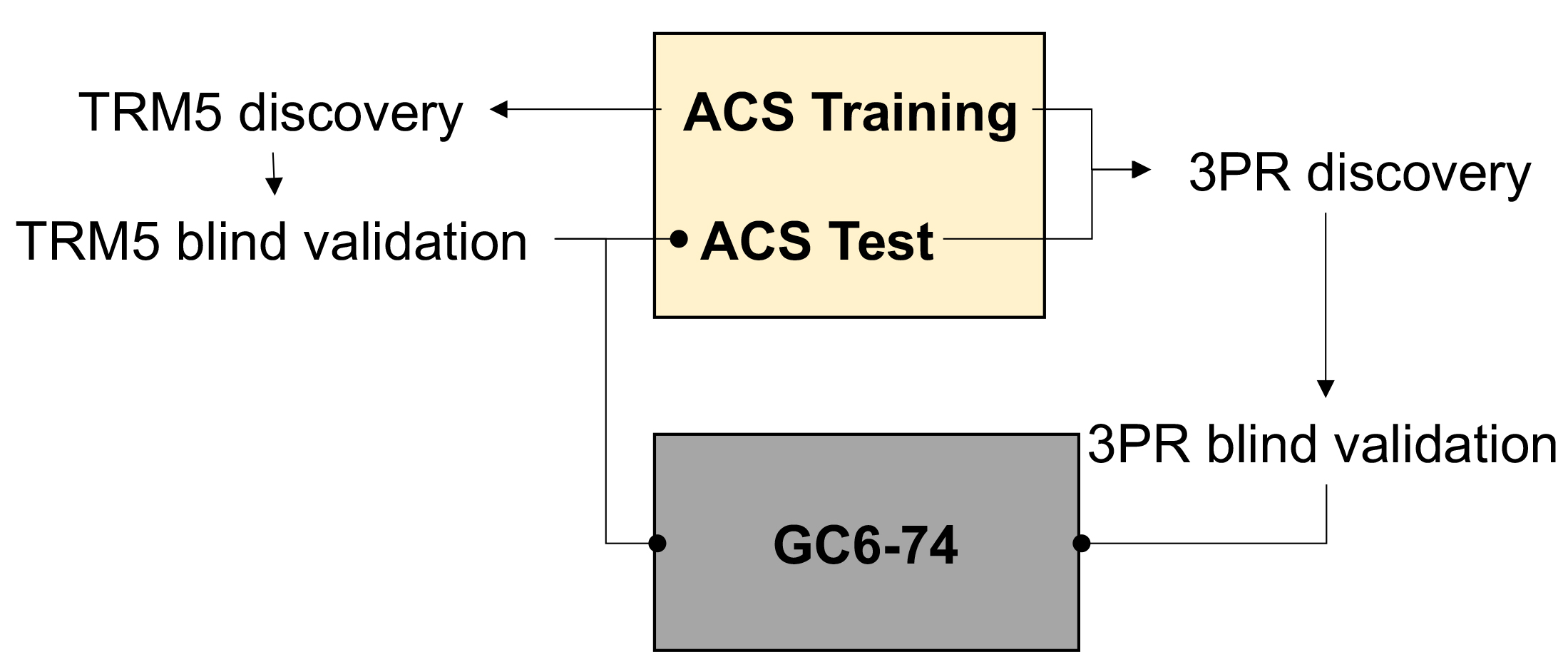

Supplement: S1 Fig — The TRM5 signature was discovered on a subset (the training set) of the ACS and then validated by blind prediction on the test set of the ACS. The 3PR signature was discovered on the full ACS set (training and test set combined). Both TRM5 and 3PR were validated by blind prediction on the GC6–74 cohort. 3PR, 3-protein pair-ratio; ACS, Adolescent Cohort Study; GC6–74, Grand Challenges 6–74; TRM5, TB Risk Model 5. (TIF) [file pmed.1002781.s003.tif]

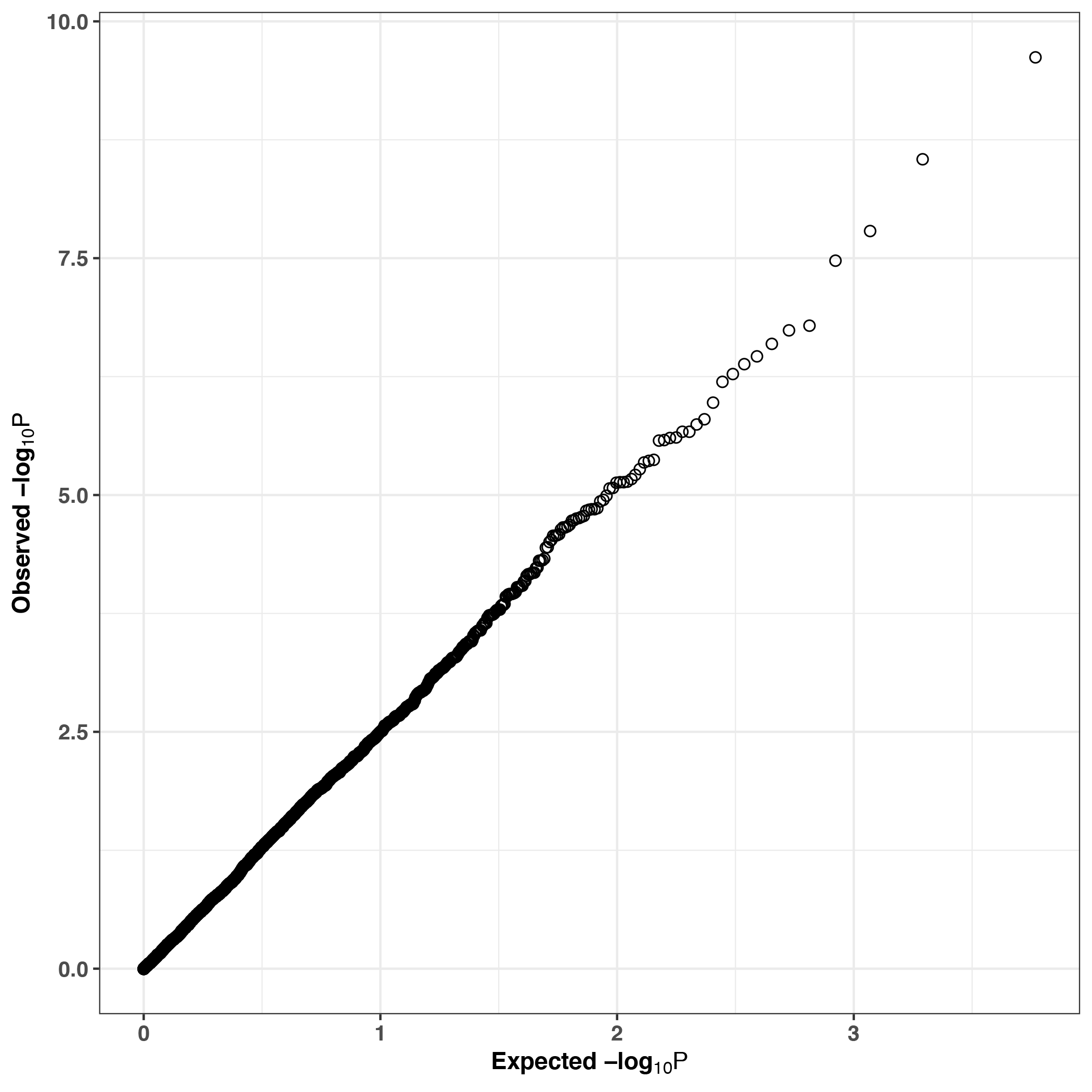

Supplement: S2 Fig — KS, Kolmogorov–Smirnov. (TIF) [file pmed.1002781.s004.tif]

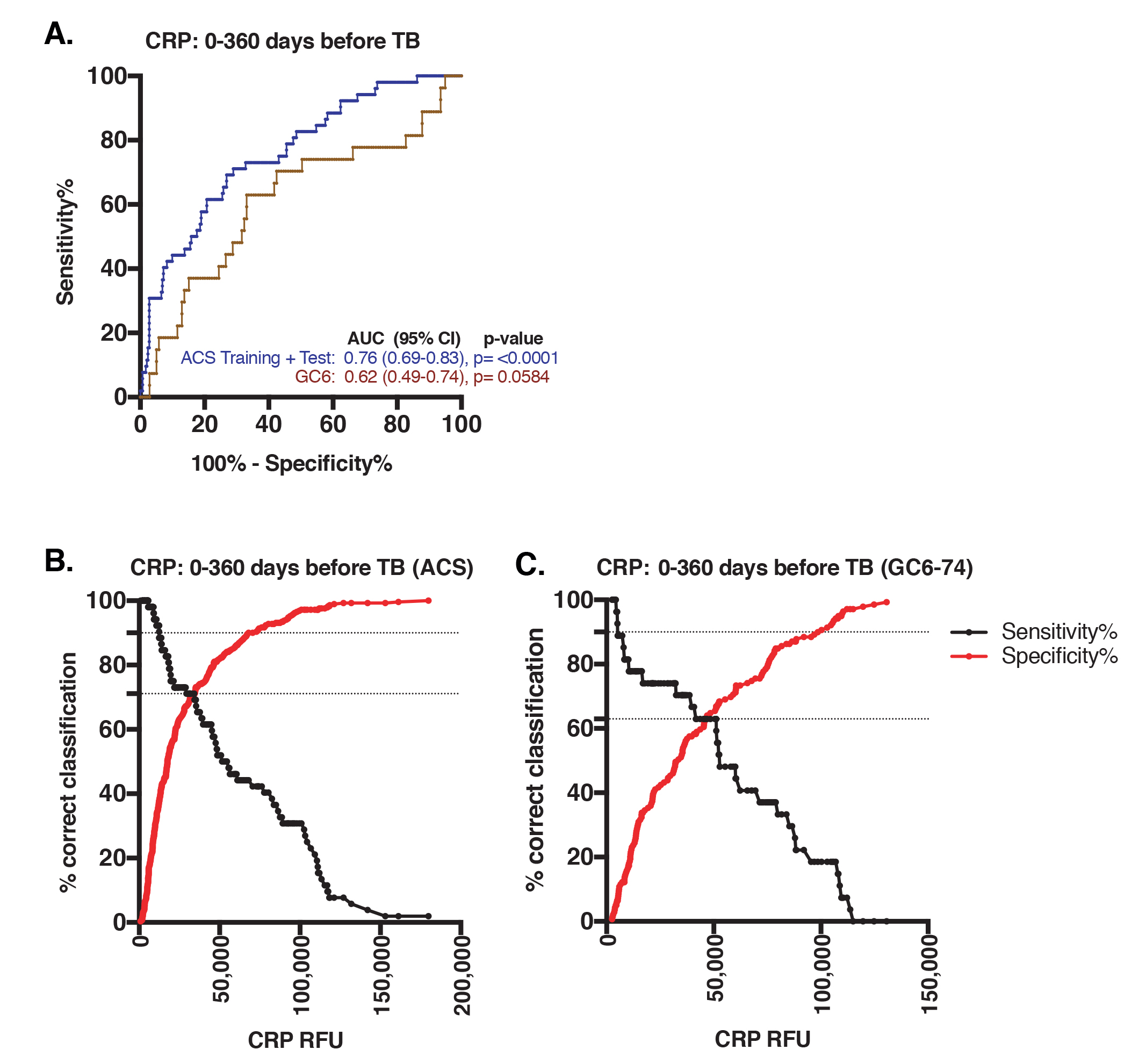

Supplement: S3 Fig — (A) ACS training and test set progressor and nonprogressor plasma samples and GC6 validation set plasma samples from time points within 1 year of TB diagnosis. Sensitivity and specificity of CRP for the (B) ACS training and test set and (C) GC6 validation set. ACS, Adolescent Cohort Study; AUC, area under the curve; CRP, C-reactive protein; GC6, Grand Challenge 6; RFU, relative fluorescence units; TB, tuberculosis. (TIF) [file pmed.1002781.s005.tif]

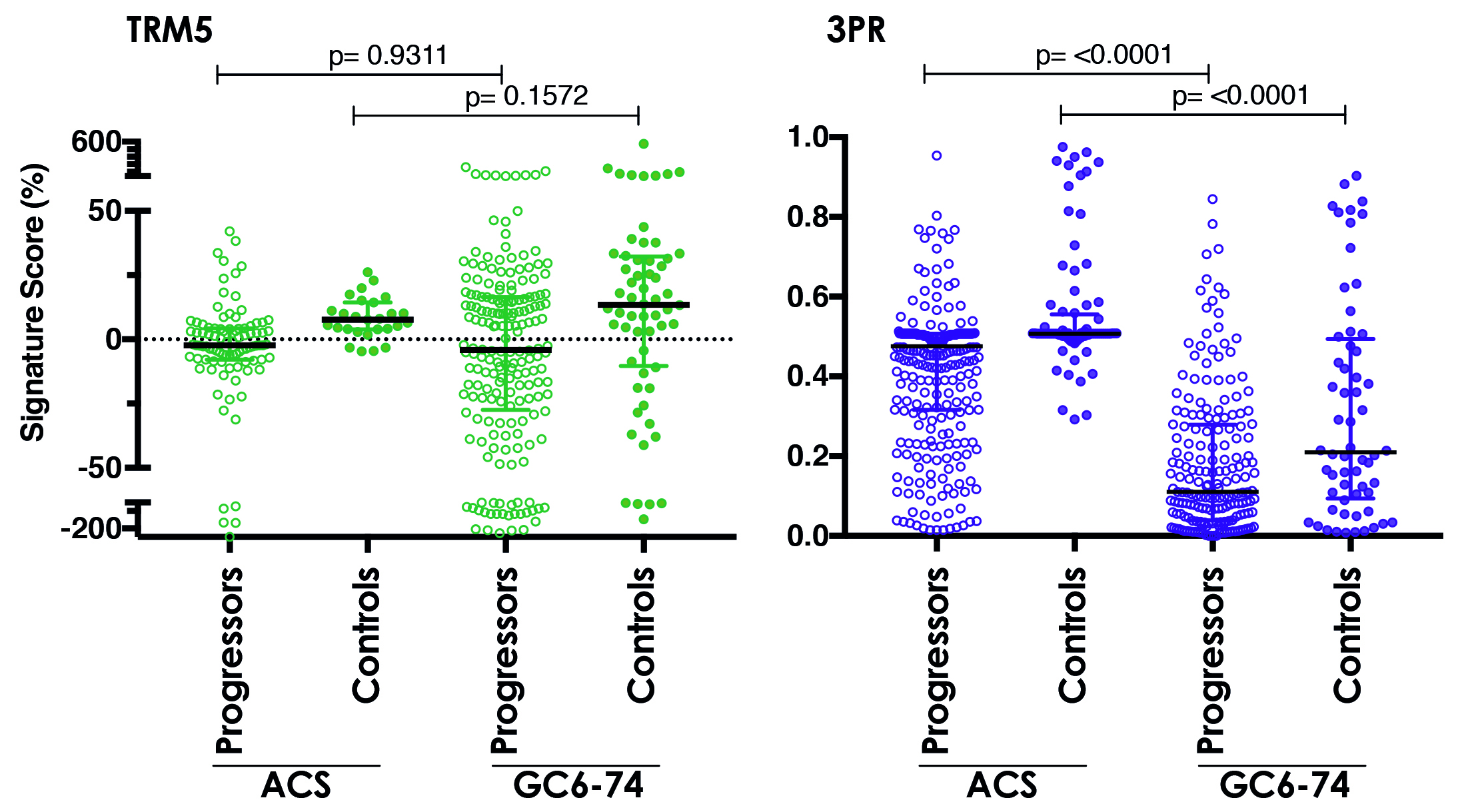

Supplement: S4 Fig — Mann–Whitney test P values are shown for comparison of each signature on different progressor and nonprogressor samples run on the different SOMAscan assays. 3PR, 3-protein pair-ratio; ACS, Adolescent Cohort Study; GC6–74, Grand Challenges 6–74; SOMAscan; TRM5, TB Risk Model 5. (TIF) [file pmed.1002781.s006.tif]
