# Supplementary material for: Discovery and validation of a prognostic proteomic signature for tuberculosis progression: A prospective cohort study
Source: PLoS Med. 2019 Apr 16;16(4):e1002781. doi: 10.1371/journal.pmed.1002781 (PMC6467365; doi:10.1371/journal.pmed.1002781)
Supplement: S1. TRIPOD checklist — (DOCX) [file pmed.1002781.s014.docx]

| **Section/Topic** | **Item** |  | **Checklist Item** | **Page** |
| --- | --- | --- | --- | --- |
| **Title and abstract** | | | | |
| Title | 1 | D;V | Identify the study as developing and/or validating a multivariable prediction model, the target population, and the outcome to be predicted. | Title page |
| Abstract | 2 | D;V | Provide a summary of objectives, study design, setting, participants, sample size, predictors, outcome, statistical analysis, results, and conclusions. | Abstract |
| **Introduction** | | | | |
| Background and objectives | 3a | D;V | Explain the medical context (including whether diagnostic or prognostic) and rationale for developing or validating the multivariable prediction model, including references to existing models. | Introduction: Paragraph 1  “Such a strategy requires prognostic tests which can accurately identify those at risk of TB disease before the onset of symptoms and further transmission” |
|  | 3b | D;V | Specify the objectives, including whether the study describes the development or validation of the model or both. | Introduction: Paragraph 3  “In this study, we proposed to identify and validate parsimonious proteomic signatures of TB disease risk.” |
| **Methods** | | | | |
| Source of data | 4a | D;V | Describe the study design or source of data (e.g., randomized trial, cohort, or registry data), separately for the development and validation data sets, if applicable. | Methods: Paragraphs 1, 2, 5 |
|  | 4b | D;V | Specify the key study dates, including start of accrual; end of accrual; and, if applicable, end of follow-up. | Methods: Paragraph 1  “Discovery: Adolescent Cohort Study (ACS) between July 6, 2005 and April 23, 2007  Methods: Paragraph 5  Validation: March 5, 2007 and October 21, 2010” |
| Participants | 5a | D;V | Specify key elements of the study setting (e.g., primary care, secondary care, general population) including number and location of centres. | Methods: Paragraphs 1,2,5  and previously described  previously described in:  Zak DE, Penn-Nicholson A, Scriba TJ, Thompson E, Suliman S, Amon LM, et al. A blood RNA signature for tuberculosis disease risk: a prospective cohort study. Lancet. 2016;387: 2312–2322.  doi:10.1016/S0140-6736(15)01316-1  Suliman S, Thompson E, Sutherland J, Weiner Rd J, Ota MOC, Shankar S, et al. Four-gene Pan-African Blood Signature Predicts Progression to Tuberculosis. Am J Respir Crit Care Med. 2018;197: 1198–1208. doi:10.1164/rccm.201711-2340OC  Mahomed H, Hawkridge T, Verver S, Geiter L, Hatherill M, Abrahams DA, et al. Predictive factors for latent tuberculosis infection among adolescents in a high-burden area in South Africa. Int J Tuberc Lung Dis. 2011;15: 331–336. Available: http://docstore.ingenta.com/cgi-bin/ds_deliver/1/u/d/ISIS/75247575.1/iuatld/ijtld/2011/00000015/00000003/art00007/6D657CA10BA54D7213771112618C404EEB43BA948A.pdf?link=http://www.ingentaconnect.com/error/delivery&format=pdf  Mahomed H, Ehrlich R, Hawkridge T, Hatherill M, Geiter L, Kafaar F, et al. TB Incidence in an Adolescent Cohort in South Africa. PLoS ONE. Public Library of Science; 2013;8: e59652. doi:10.1371/journal.pone.0059652” |
|  | 5b | D;V | Describe eligibility criteria for participants. | Methods: Paragraph 1, 2, 5  and previously described |
|  | 5c | D;V | Give details of treatments received, if relevant. | N/A |
| Outcome | 6a | D;V | Clearly define the outcome that is predicted by the prediction model, including how and when assessed. | Methods: Paragraph 9, 10,  And S2 Text  “Blinding procedure: Samples from the ACS test set (33% of the progressor and non-progressor cohort) were blinded through non-sequential randomly generated codes, held in a locked database by the project manager. Unblinding occurred in a staged manner; once models and scripts were locked down, and each partner institute had validated that results obtained on the blinded set were identical and reproducible, an interim analysis of longitudinally collected samples from the same participants were performed, without revealing case/control status. Subsequently, progressor and non-progressor status were unblinded to all sites simultaneously, and performance of models independently calculated and confirmed.  All 254 GC6-74 plasma samples were de-identified and provided non-sequential randomly generated codes, which were held in a locked database by the project manager. Unblinding of samples, matched participants and progressor/non-progressor status occurred simultaneously. A detailed description of the analysis strategy for signature discovery, verification and validation is available in the Supplement.” |
|  | 6b | D;V | Report any actions to blind assessment of the outcome to be predicted. | Methods: Paragraph 9, 10,  And S2 Text  “Blinding procedure…” |
| Predictors | 7a | D;V | Clearly define all predictors used in developing or validating the multivariable prediction model, including how and when they were measured. | Methods: Paragraph 11-14    “Statistical analysis and model development:” |
|  | 7b | D;V | Report any actions to blind assessment of predictors for the outcome and other predictors. | Methods: Paragraph 9, 10,  And S2 Text  “Blinding procedure: Samples from the ACS test set (33% of the progressor and non-progressor cohort) were blinded through non-sequential randomly generated codes, held in a locked database by the project manager. Unblinding occurred in a staged manner; once models and scripts were locked down, and each partner institute had validated that results obtained on the blinded set were identical and reproducible, an interim analysis of longitudinally collected samples from the same participants were performed, without revealing case/control status. Subsequently, progressor and non-progressor status were unblinded to all sites simultaneously, and performance of models independently calculated and confirmed.  All 254 GC6-74 plasma samples were de-identified and provided non-sequential randomly generated codes, which were held in a locked database by the project manager. Unblinding of samples, matched participants and progressor/non-progressor status occurred simultaneously. A detailed description of the analysis strategy for signature discovery, verification and validation is available in the Supplement.“ |
| Sample size | 8 | D;V | Explain how the study size was arrived at. | Results: Paragraph 1, 2  “Sample availability and distribution.  Plasma samples were available for 37 progressors and 106 non-progressors from the Adolescent Cohort Study….  Similarly, plasma samples from 34 progressors and 115 non-progressors from the Gambian GC6-74 cohort were available for blind validation and distributed between 1-24 months before TB diagnosis” |
| Missing data | 9 | D;V | Describe how missing data were handled (e.g., complete-case analysis, single imputation, multiple imputation) with details of any imputation method. | N/A |
| Statistical analysis methods | 10a | D | Describe how predictors were handled in the analyses. | Methods: Paragraph 11-14  “Statistical analysis and model development:” |
|  | 10b | D | Specify type of model, all model-building procedures (including any predictor selection), and method for internal validation. | Methods: Paragraph 11-14,  And S2 text  “Statistical analysis and model development:” |
|  | 10c | V | For validation, describe how the predictions were calculated. | Methods: Paragraph 9, 10,  and S2 Text    “Blinding procedure: Samples from the ACS test set (33% of the progressor and non-progressor cohort) were blinded through non-sequential randomly generated codes, held in a locked database by the project manager. Unblinding occurred in a staged manner; once models and scripts were locked down, and each partner institute had validated that results obtained on the blinded set were identical and reproducible, an interim analysis of longitudinally collected samples from the same participants were performed, without revealing case/control status. Subsequently, progressor and non-progressor status were unblinded to all sites simultaneously, and performance of models independently calculated and confirmed.  All 254 GC6-74 plasma samples were de-identified and provided non-sequential randomly generated codes, which were held in a locked database by the project manager. Unblinding of samples, matched participants and progressor/non-progressor status occurred simultaneously. A detailed description of the analysis strategy for signature discovery, verification and validation is available in the Supplement.“ |
|  | 10d | D;V | Specify all measures used to assess model performance and, if relevant, to compare multiple models. | Methods: Paragraph 11-14,  And S2 text  “Statistical analysis and model development:” |
|  | 10e | V | Describe any model updating (e.g., recalibration) arising from the validation, if done. | N/A |
| Risk groups | 11 | D;V | Provide details on how risk groups were created, if done. | N/A |
| Development vs. validation | 12 | V | For validation, identify any differences from the development data in setting, eligibility criteria, outcome, and predictors. | Table 1 |
| **Results** | | | | |
| Participants | 13a | D;V | Describe the flow of participants through the study, including the number of participants with and without the outcome and, if applicable, a summary of the follow-up time. A diagram may be helpful. | Methods: Paragraphs 1,2,5,  Table 1, Table 2,  and previously described,  Previously described in:  Zak DE, Penn-Nicholson A, Scriba TJ, Thompson E, Suliman S, Amon LM, et al. A blood RNA signature for tuberculosis disease risk: a prospective cohort study. Lancet. 2016;387: 2312–2322.  doi:10.1016/S0140-6736(15)01316-1  Suliman S, Thompson E, Sutherland J, Weiner Rd J, Ota MOC, Shankar S, et al. Four-gene Pan-African Blood Signature Predicts Progression to Tuberculosis. Am J Respir Crit Care Med. 2018;197: 1198–1208. doi:10.1164/rccm.201711-2340OC  Mahomed H, Hawkridge T, Verver S, Geiter L, Hatherill M, Abrahams DA, et al. Predictive factors for latent tuberculosis infection among adolescents in a high-burden area in South Africa. Int J Tuberc Lung Dis. 2011;15: 331–336. Available: http://docstore.ingenta.com/cgi-bin/ds_deliver/1/u/d/ISIS/75247575.1/iuatld/ijtld/2011/00000015/00000003/art00007/6D657CA10BA54D7213771112618C404EEB43BA948A.pdf?link=http://www.ingentaconnect.com/error/delivery&format=pdf  Mahomed H, Ehrlich R, Hawkridge T, Hatherill M, Geiter L, Kafaar F, et al. TB Incidence in an Adolescent Cohort in South Africa. PLoS ONE. Public Library of Science; 2013;8: e59652. doi:10.1371/journal.pone.0059652 |
|  | 13b | D;V | Describe the characteristics of the participants (basic demographics, clinical features, available predictors), including the number of participants with missing data for predictors and outcome. | Table 1, Table 2 |
|  | 13c | V | For validation, show a comparison with the development data of the distribution of important variables (demographics, predictors and outcome). | Table 1 |
| Model development | 14a | D | Specify the number of participants and outcome events in each analysis. | Table 1, Table 2 |
|  | 14b | D | If done, report the unadjusted association between each candidate predictor and outcome. | Table 2 |
| Model specification | 15a | D | Present the full prediction model to allow predictions for individuals (i.e., all regression coefficients, and model intercept or baseline survival at a given time point). | S3-S7 Tables, |
|  | 15b | D | Explain how to the use the prediction model. | Methods: Paragraph 4, 11  S2 Text  Statistical analysis and model development.  “The script for computing the TRM5 signature is available from SomaLogic upon request. The script for computing for the 3PR signature is available at BitBucket (https://bitbucket.org/satvi/3pr).” |
| Model performance | 16 | D;V | Report performance measures (with CIs) for the prediction model. | Table 2, Fig 2, Fig 3, Fig 5 |
| Model-updating | 17 | V | If done, report the results from any model updating (i.e., model specification, model performance). | N/A |
| **Discussion** | | | | |
| Limitations | 18 | D;V | Discuss any limitations of the study (such as nonrepresentative sample, few events per predictor, missing data). | Abstract: Methods and Findings,  and Discussion: Paragraph 7  “Our study had a number of limitations. Greater statistical power for signature discovery” |
| Interpretation | 19a | V | For validation, discuss the results with reference to performance in the development data, and any other validation data. | Discussion: Paragraph 1, 5  “Using a well-characterised, prospective longitudinal cohort of M. tuberculosis-infected South African adolescents, we discovered two prognostic protein signatures, TRM5 and 3PR, that successfully identified individuals at risk of incident TB disease risk within a year of the onset of disease symptoms. Validation of the prognostic performance of these signatures in an independent cohort of household contacts of TB patients from The Gambia represents a first step to an affordable and practical prognostic biomarker for TB  We observed a systematic shift in signal magnitudes generated by the validation assay, compared to the >3000-plex discovery assay.” |
|  | 19b | D;V | Give an overall interpretation of the results, considering objectives, limitations, results from similar studies, and other relevant evidence. | Results: Paragraph 11  “Neither TRM5 nor 3PR achieved the minimum criteria for an incipient TB test set out by by FIND and WHO [19] and it is clear that more work is needed to improve the performance of prognostic signatures based on proteins. The same was true of the prognostic performance of CRP. Notably, a recent diagnostic accuracy study conducted in two Ugandan HIV/AIDS clinics showed that point-of-care CRP screening of HIV-infected people with CD4 counts <351 cells per μL who were initiating antiretroviral therapy yielded 89% sensitivity and 72% specificity for culture confirmed TB [25]. The study supported use of CRP as a TB screening test to improve efficiency of case finding.  Nevertheless, our study reports, to the best of our knowledge, the first proteomic prognostic signature for TB and demonstrates feasibility of the approach.” |
| Implications | 20 | D;V | Discuss the potential clinical use of the model and implications for future research. | Results: Paragraph 11 |
| **Other information** | | | | |
| Supplementary information | 21 | D;V | Provide information about the availability of supplementary resources, such as study protocol, Web calculator, and data sets. | Throughout Narrative, and within S2 Text |
| Funding | 22 | D;V | Give the source of funding and the role of the funders for the present study. | At request of editor, removed from main narrative and added ony to online submission field |

*Items relevant only to the development of a prediction model are denoted by D, items relating solely to a validation of a prediction model are denoted by V, and items relating to both are denoted D;V. We recommend using the TRIPOD Checklist in conjunction with the TRIPOD Explanation and Elaboration document.
