# Supplementary figures and images for: Correction: Discovery and validation of a prognostic proteomic signature for tuberculosis progression: A prospective cohort study
Source: PLoS Med. 2019 Jul 18;16(7):e1002880. doi: 10.1371/journal.pmed.1002880 (PMC6638739; doi:10.1371/journal.pmed.1002880)

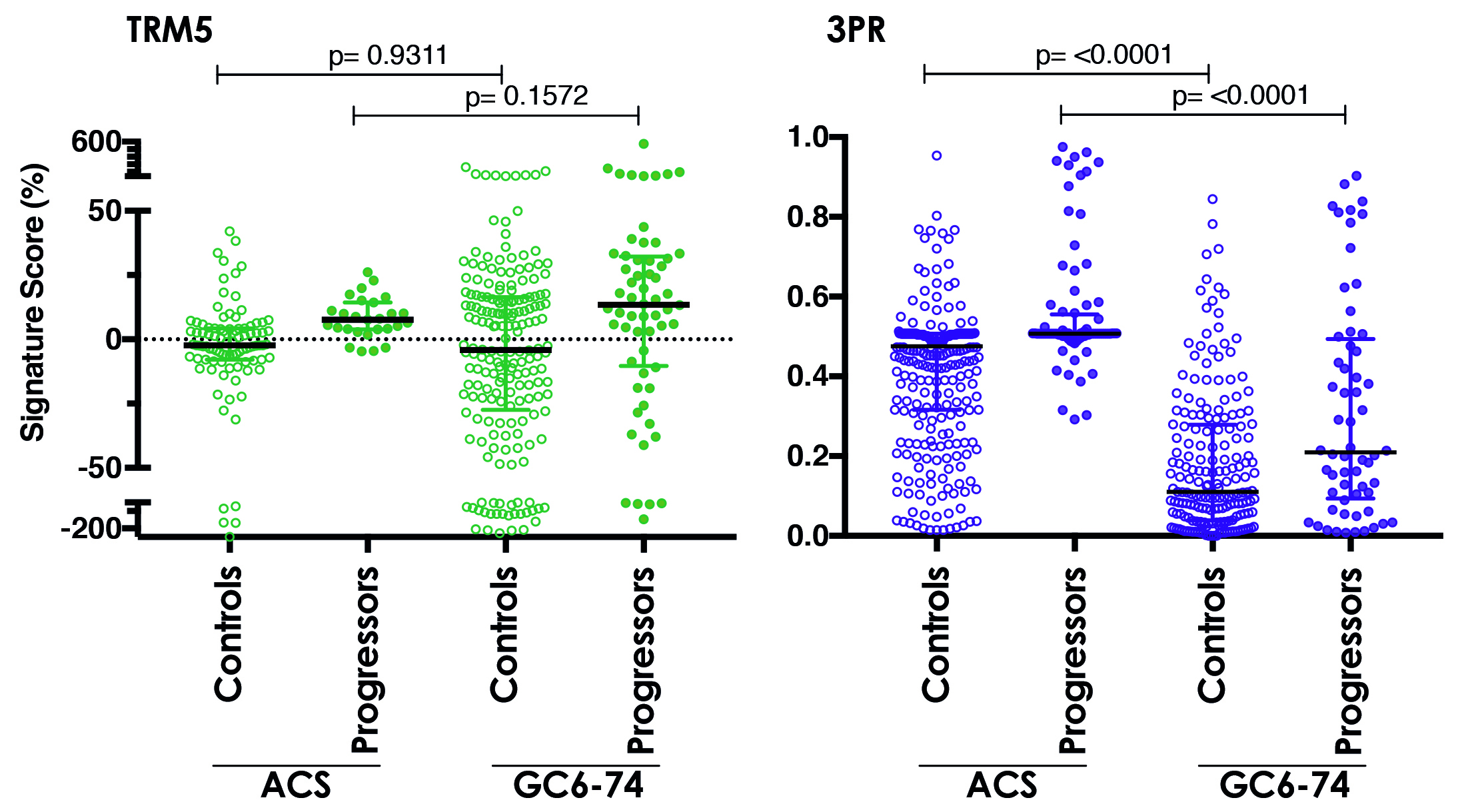

Supplement: S4 Fig — Mann–Whitney test P values are shown for comparison of each signature on different progressor and nonprogressor samples run on the different SOMAscan assays. 3PR, 3-protein pair-ratio; ACS, Adolescent Cohort Study; GC6–74, Grand Challenges 6–74; SOMAscan; TRM5, TB Risk Model 5. (TIF) [file pmed.1002880.s001.tif]
